# Self-inflicted DNA double-strand breaks sustain tumorigenicity and stemness of cancer cells

**DOI:** 10.1038/cr.2017.41

**Published:** 2017-03-24

**Authors:** Xinjian Liu, Fang Li, Qian Huang, Zhengxiang Zhang, Ling Zhou, Yu Deng, Min Zhou, Donald E Fleenor, He Wang, Michael B Kastan, Chuan-Yuan Li

**Affiliations:** 1 Department of Dermatology, Duke University Medical Center, Durham, NC 27710, USA; 2 Cancer Center, Shanghai General Hospital, School of Medicine, Shanghai Jiaotong University, Shanghai 201620, China; 3 Department of Surgery, Shanghai General Branch Hospital, School of Medicine, Shanghai Jiaotong University, Shanghai 200081, China; 4 State Key Laboratory of Oncogenes and Related Genes, Shanghai Cancer Institute, Shanghai JiaotongUniversity, Shanghai 200032, China; 5 Department of Pharmacology and Cancer Biology, Duke University Medical Center, Durham, NC 27710, USA; 6 West China Second University Hospital, Sichuan University, Chengdu 610041, China; 7 Duke Cancer Institute, Duke University Medical Center, Durham, NC 27710, USA

**Keywords:** sublethal caspase activation, spontaneous DNA double-strand breaks, DNA damage response, ATM activation, cancer stem cells

## Abstract

DNA double-strand breaks (DSBs) are traditionally associated with cancer through their abilities to cause chromosomal instabilities or gene mutations. Here we report a new class of self-inflicted DNA DSBs that can drive tumor growth irrespective of their effects on genomic stability. We discover a mechanism through which cancer cells cause DSBs in their own genome spontaneously independent of reactive oxygen species or replication stress. In this mechanism, low-level cytochrome c leakage from the mitochondria leads to sublethal activation of apoptotic caspases and nucleases, which causes DNA DSBs. In response to these spontaneous DNA DSBs, ATM, a key factor involved in DNA damage response, is constitutively activated. Activated ATM leads to activation of transcription factors NF-κB and STAT3, known drivers of tumor growth. Moreover, self-inflicted DNA DSB formation and ATM activation are important in sustaining the stemness of patient-derived glioma cells. In human tumor tissues, elevated levels of activated ATM correlate with poor patient survival. Self-inflicted DNA DSBs therefore are functionally important for maintaining the malignancy of cancer cells.

## Introduction

DNA double-strand breaks (DSBs) are a form of DNA damage that if left unrepaired, can have serious consequences such as chromosomal aberrations or cell death. Therefore, cells have a sophisticated system to detect and repair DSBs. DSBs can be caused by exogenous factors such as ionizing radiation or endogenous stress such as reactive oxygen species. Repairing of DSBs in mammalian cells is a multi-step process that includes sensing and detection of the DSBs, causing cell cycle arrest, repairing of the DSBs, and restoration of cell cycle progression. The entire process of cellular response to DSBs is commonly called the DNA damage response (DDR)^1^.

The eukaryotic DNA DSB repair system includes two distinct mechanisms: homologous recombination (HR)^2^ and non-homologous end joining (NHEJ)^3^. In HR, the cell detects the DSB, finds a homologous DNA sequence, and uses it as a template to finish DSB repair. In NHEJ, the cell detects the DSB and directly ligates the broken ends together to repair the DSB. In general, HR is more accurate as it usually copies the missing sequence from an intact, homologous DNA template. NHEJ, on the other hand, often leads to insertions or deletions because it does not use a template. In mammalian cells, two important phosphoinositide (PI) 3-kinase family proteins are involved in the detection of DSBs: ATM (ataxia telangiectasia mutated)^4,5,6,7^ and DNA-PKcs^8^. DSB detection by ATM can lead to activation of either HR- or NHEJ-mediated repair. DSB detection by DNA-PKcs, on the other hand, leads to activation of NHEJ exclusively.

Because unrepaired DSBs lead to genetic instability and carcinogenesis, repairing them is critical for the survival of the whole organism^9^. Indeed, mammalian cellular DDR system plays key roles in the prevention of carcinogenesis. Among the many DDR proteins, ATM was shown to play a pivotal role in suppressing cancer development^10^. ATM mutations have been implicated in leukemia^11^, lymphoma^12^, and breast cancer^13^. Moreover, DSBs and ATM activation are often observed in pre-cancerous lesions and serves as a barrier for oncogenesis^14,15,16^. In those cases, DSBs are attributed to oncogene-induced DNA replication stress. Replication stress-induced DSBs are shown to cause activation of ATM, which leads to cell cycle arrest, cellular senescence, or apoptosis. Attenuation of DDR by ATM inhibition leads to an increased rate of oncogenic transformation.

Aside from detecting and repairing of DSBs that are passively incurred (for example by ionizing radiation exposure), DSB repair factors are also involved in cases where DSBs are actively induced in mammalian cells. For example, in VDJ recombination of B-cells or T-cells, DSBs are actively induced and repaired by the NHEJ process. The importance of the NHEJ-mediated repair was demonstrated by the fact that mutations in the DNA-PKcs gene lead to severe combined immunodeficiency with deficiencies in both B- and T-cells^17,18,19^.

Here we present evidence that many tumor cells possess spontaneously arising DNA DSBs induced by sublethal activation of apoptotic caspases and DNA nucleases in the absence of external stress. Most unexpectedly, the self-inflicted DSBs lead to persistent and robust activation of ATM that sustains tumorigenicity and cancer cell stemness instead of suppressing them.

## Results

### Observation of spontaneous DNA damage foci formation in cancer cells

**Figure 1:**
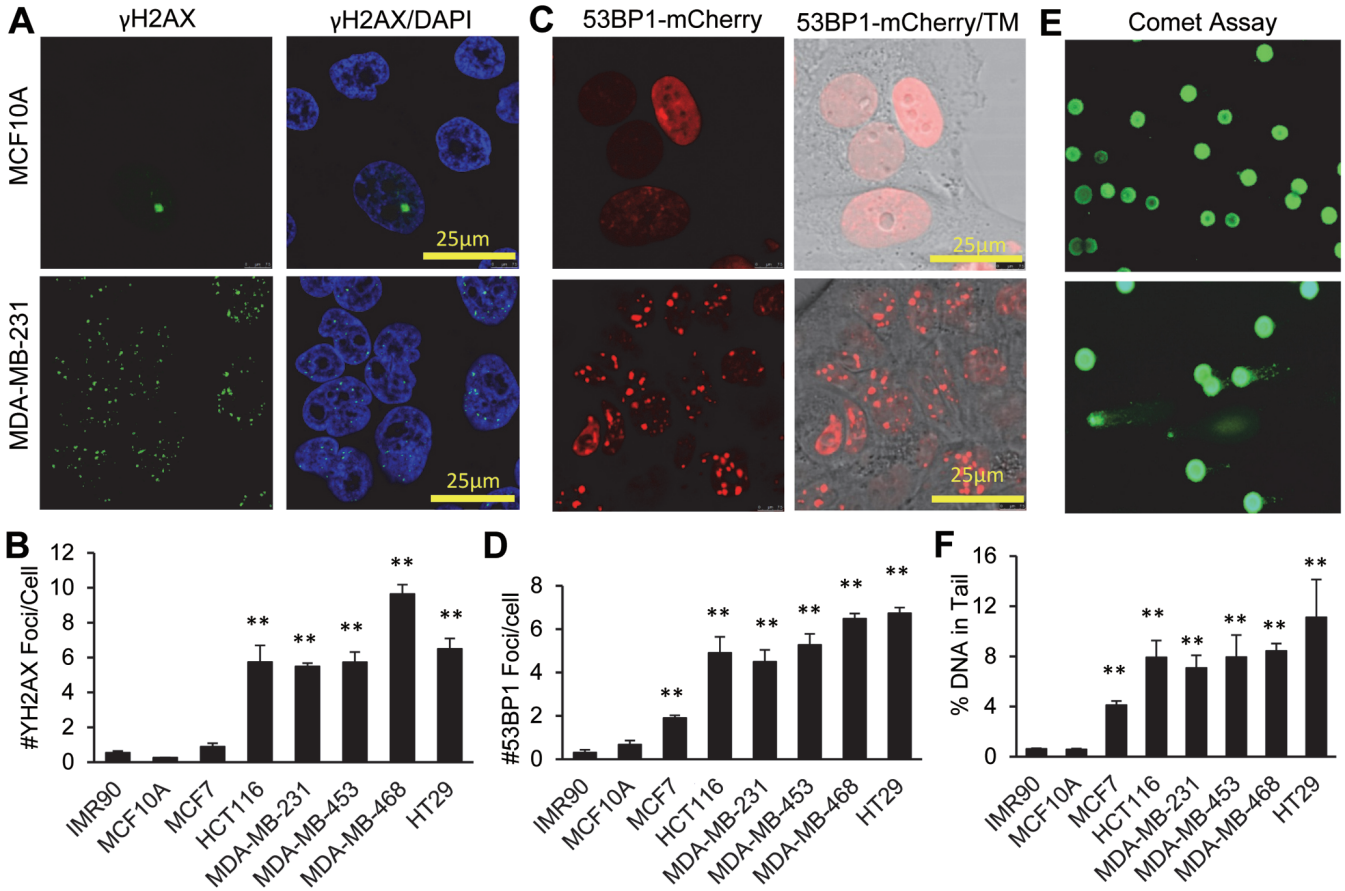
Spontaneous DNA double-strand breaks (spDSBs) in cancer cells. **(A)** Representative images of γH2AX foci in MCF10A (top panels) and MDA-MB-231(lower panels) cells. **(B)** Quantitative analysis of γH2AX foci in non-transformed (IMR90 and MCF10A) and transformed (all others) cells. **(C)** Representative images of 53BP1 foci in MCF10A and MDA-MB-231 cells. **(D)** Quantitative analysis of 53BP1 foci in non-transformed (IMR90 and MCF10A) and transformed (all others) cells. **(E)** Representative images of comet assays performed in MCF10A and MDA-MB-231 cell lines. **(F)** Quantitative results of comet assay for non-transformed cells (IMR90 and MCF10A) and cancer cells. Error bars in **B**, **D**, and **F** represent SEM. ^**^P < 0.001 (*n* = 3, Student's *t*-test) when comparing the indicated cancer cells with IMR90 cells. TM, transmitted white light.

A systematic investigation of γH2AX foci was carried out in a panel of transformed and non-transformed human cells. γH2AX is a well-recognized marker for DSBs in mammalian cells^20^. We synchronized the cells in G1 phase to exclude the counting of γH2AX foci often encountered in S phase of the cell cycle. We validated our serum starvation-based synchronization protocol by use of flow cytometry analysis (Supplementary information, Figure S1A), cyclin D1 immunofluorescence staining, and EdU (5-ethynyl-2′dexouridine) labeling (Supplementary information, Figure S1B). Compared with a primary fibroblast cell strain (IMR90) and an immortalized but untransformed cell line (MCF10A), the tumor lines (HCT116, MDA-MB-231, MDA-MB-453, MDA-MB-468, and HT29) have much higher levels of background γH2AX foci (Figure 1A and 1B). The authenticity of the γH2AX in representing DSBs was further verified by the detection of 53BP1 foci through the use of a non-invasive 53BP1-mCherry reporter^21^ (Figure 1C and 1D). Furthermore, by use of cyclin D1 staining and EdU labeling (Supplementary information, Figure S1C and S1D), we confirmed that cells in the G1 phase of the cell cycle possessed robust levels of γH2AX foci, thereby demonstrating the existence of DNA DSBs independent of replication stress that were reported previously^15,16^. Using marker co-staining, we also confirmed the existence of γH2AX foci in G1 phase in exponentially growing cells in normal serum conditions (Supplementary information, Figure S1E), thereby excluding stress from serum starvation itself as a potential confounding factor in inducing DNA damage foci.

By use of flow cytometry, cells with different levels of 53BP1-mCherry could be sorted into different groups (Supplementary information, Figure S1F). Cells with high (R5 group) and low (R1 group) reporter activities possessed high and low γH2AX foci levels, respectively (Supplementary information, Figure S1G and S1H). Most importantly, when both γH2AX and 53BP1 foci were counted in the same samples (Supplementary information, Figure S1I), they showed close to 90% concordance (Supplementary information, Figure S1H and S1I).

To further confirm the presence of self-inflicted DNA DSBs in cancer cells, the comet assay, which is a commonly used DNA damage assay independent of the DNA damage foci assays described above, was used to measure the levels of cellular DNA DSBs^22^. Our data showed the presence of DNA in the “comet tails” of the cells run in agarose gels (Figure 1E and 1F). The percentages of DNA in the tails, an indication of DNA damage, were consistent with the trends observed for 53BP1 and γH2AX foci in the cells. Furthermore, when we sorted MDA-MB-231 cells according their 53BP1-mCherry reporter levels, those cells with higher levels of 53BP1-mCherry fluorescence showed higher levels of DNA in their comet tails (Supplementary information, Figure S1J). These data confirmed the concordance of the 53BP1-reporter with the comet assay. Therefore, our data demonstrate the robust presence of DSBs in different unstressed cancer cells by use of three different assays.

Our results are therefore consistent with earlier results demonstrating the existence of DNA DSBs in precancerous lesions and some cancer cells^15,16^. For the rest of this report, we intend to call these DSBs spontaneous DSBs (spDSBs) to distinguish them from traditional, DNA damaging agent-induced DSBs.

### The source of spDSBs in cancer cells

One major issue in understanding spontaneously arising DSBs in cancer cells is their origin. Previously, replication stress has been identified as potential causes of DSBs for some cancer cells^15,16^. However, because of the extensive nature of spDSBs observed, we hypothesized that sublethal activation of apoptotic caspases and nucleases caused by “leaky” mitochondria in cancer cells could be another source of these spDSBs. Previously, we have observed sublethal activation of caspases in induction of induced pluripotent stem cells^23^. More recently, we and others showed that mammalian cells exposed to external stress can survive limited mitochondrial membrane leakage and caspase activation, which could induce *de novo* DNA DSBs independent of initial stress-induced DNA damage^24,25,26^.

**Figure 2:**
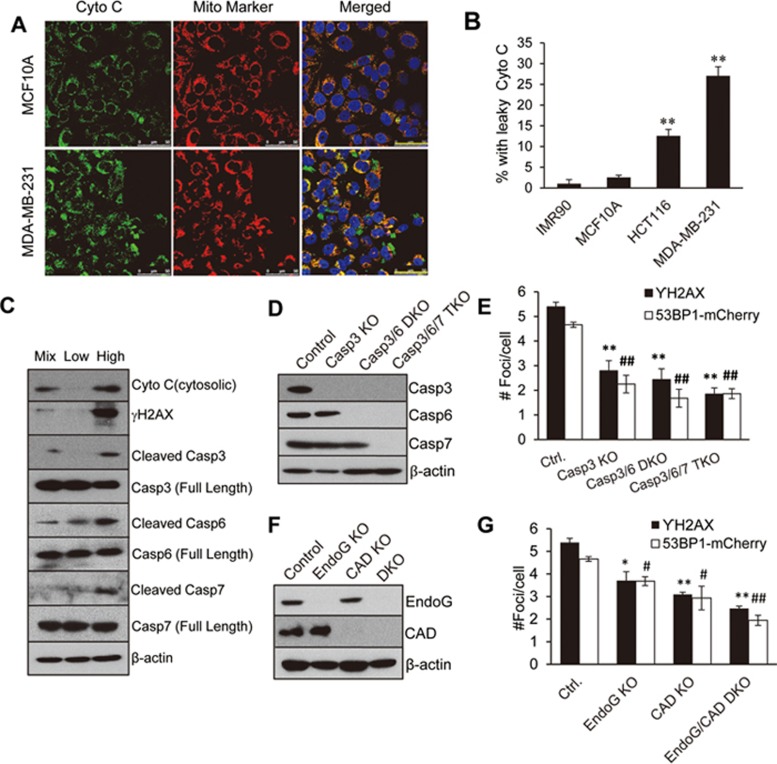
Spontaneous, sublethal activation of apoptotic caspases (-3, -6, and -7) and endonucleases (CAD and EndoG) as a major source of spDSBs in cancer cells. **(A)** Confocal microscope imaging of endogenous cytochrome c in MCF10A (top panels) and MDA-MB-231 (lower panels) cells. A mitochondrial (Mito) marker antibody was used to stain for mitochondria. Cytochrome c staining outside the mitochondria in MDA-MB-231 tumor cells is clearly visible in the merged panels. Scale bars represent 20 μm. **(B)** Percentage of cells with “leaky” extra-mitochondrial expression of cytochrome *c*. Error bars represent SEM. ^**^P < 0.001, when comparing the tumor cells vs IMR90 primary fibroblast cells (Student's *t*-test; *n* = 3). **(C)** Western blot analysis of cytosolic cytochrome *c* (Cyto c), γH2AX, and full-length and cleaved caspase-3,-6, or -7 in unsorted (Mix) and sorted MDA-MB-231 cells with low and high levels of 53BP1-mCherry reporter activities. See Supplementary information, Figure S1F (R5 or R1) for criteria for 53BP1-mCherry-high or -low cells. **(D)** Western blot analysis of caspase expression in CRISPR/Cas9-mediated *caspase-3* knockout (Casp3 KO), *caspase-3* and *caspase-6* double-knockout (Casp3/6 DKO), and *caspase-3*, *caspase-6* and *caspase-7* triple knockout (Casp3/6/7 TKO) MDA-MB-231 cells. **(E)** Average number of γH2AX and 53BP1-mCherry foci in control and *caspase-3*, -*6*, and -*7* single, double, and triple knockout MDA-MB-231 cells. **(F)** Western blot analysis protein expression in CRISPR-/Cas9-mediated *EndoG* knockout (EndoG KO), *CAD* knockout (CAD KO), and *EndoG* and *CAD* double-knockout (EndoG/CAD DKO) MDA-MB-231 cells. **(G)** Average number of γH2AX and 53BP1-mCherry foci in control and EndoG KO, CAD KO, and EndoG/CAD DKO MDA-MB-231 cells. Error bars in **E** and **G** represent SEM (*n* = 3). Ctrl. vs respective γH2AX groups: ^*^P < 0.05; ^**^P < 0.001. Ctrl. vs other 53BP1-mCherry group: ^#^*P* < 0.05; ^##^*P* < 0.001.

In order to test our hypothesis, we carried out immunofluorescence staining of cytochrome c. Non-oncogenic cells (IMR90 and MCF10A) exhibited very low levels of extra-mitochondrial cytochrome c leakage (< 5% of examined cells; Figure 2A and 2B). In comparison, two tumor cell lines (HCT116 and MDA-MB-231) have significant extra-mitochondrial cytochrome c leakage (15%-30% of examined cells; Figure 2A and 2B). Furthermore, overexpression of BCL-xL in MDA-MB-231 cells reduced the fraction of cells with extra-mitochondrial cyto c (Supplementary information, Figure S2A). Similar observation was made in MDA-MB-231 cells with shRNA-mediated double knockdown of Bax/Bak (Supplementary information, Figure S2A). Western blot analysis of the cytoplasmic fraction of cellular lysates confirmed these observations of cytochrome c leakage (Supplementary information, Figure S2B; see Supplementary information, Table S1 for antibody information) in tumor cells.

In order to evaluate the relationships among cytochrome c leakage, caspase activation and spDSB formation, we carried out western blot analysis in reporter-transduced, non-sorted, and sorted MDA-MB-231 cells. Cells with higher levels of 53BP1-mCherry reporter activities (and therefore higher number of DSBs) exhibited higher levels of cytochrome c leakage, higher levels γH2AX, and higher levels of cleaved caspases-3, -6, and -7 than the non-sorted cells (labeled as Mix) or sorted cells with low 53BP1-mCherry reporter activities (Figure 2C).

We next attempted to establish whether there is a causal relationship between caspase activation and spDSB formation. By use of the CRISPR technology, we generated MDA-MB-231 cells with genetic ablation of caspase-3 (Casp3 KO), caspase-3+caspase-6 (Casp3/6 DKO), or caspase-3+caspase-6+caspase-7 (Casp3/6/7 TKO) genes (Figure 2D; see also Supplementary information, Tables S2 and S3 for sgRNA sequence information and sequencing confirmation of knockouts, respectively). Knockout of the caspase genes had minimal effects on cellular growth rate (Supplementary information, Figure S2C). In fact, the doubling times for vector control, Casp3 KO, Casp3/6 DKO, and Casp3/6/7 TKO cells are 33, 34, 32, and 32 h, respectively. These cells were then evaluated for their DNA damage foci numbers. Our results showed that caspase-3 deficiency significantly reduced spDSB formation in MDA-MB-231 cells. Further knockout of caspase-6 reduced spDSB formation even more, suggesting some redundancy in inducing DSBs by the apoptotic caspases. Cells with triple knockout of caspase-3/6/7 further reduced the number of DSB foci per cell. However, the reduction over the double-knockout cells was small (Figure 2E).

A causal role of caspase-3 in inducing spDSBs was further shown when comparing wild-type MCF7 cells with those of MCF7-Casp3 cells (Supplementary information, Figure S2D). MCF7 cells are deficient in caspase-3 expression. MCF7-Casp3 cells have artificially transduced, constitutively expressed caspase-3. Consistent with the role of caspase-3 in inducing spDSBs, γH2AX and 53BP1-mCherry foci numbers were significantly increased in these cells compared with parental MCF7 cells (Supplementary information, Figure S2E), thereby confirming a causative role for caspase-3 in spDSB induction in MCF7 cells.

What are the downstream factors that cause DSB formation? We hypothesized that two well-established apoptotic nucleases: endonuclease G (EndoG) and caspase-dependent DNase (CAD) may be responsible for causing DSB formation in host cells. Compared with IMR90 primary fibroblast cells, most cancer cells showed enhanced cleavage of ICAD (Supplementary information, Figure S2F), which is an inhibitor of CAD and is normally intact^27,28^. ICAD cleavage is an indication of CAD activation. Western blot analysis in MCF7 cells with or without caspase-3 expression and in MDA-MB-231 cells with various combinations of caspase knockout showed a clear relationship between executioner caspases (-3, -6, and -7) and ICAD cleavage (Supplementary information, Figure S2G).

We next analyzed EndoG location in various normal and cancer cells. EnodG normally resides within the mitochondria but migrates into the nucleus during apoptosis^29^. Western blot analysis showed clear spontaneous EndoG translocation into the nucleus from the mitochondria in various cancer cells. In comparison, the primary fibroblast IMR90 cells showed significantly less EndoG translocation (Supplementary information, Figure S2H). Western blot analysis in MCF7 cells with or without Casp3 expression and in MDA-MB-231 cells with various combinations of caspase knockouts showed a clear relationship between executioner caspases (-3, -6, and -7) and the levels of nuclear EndoG (Supplementary information, Figure S2I). Therefore, our data support the possibility that both apoptotic nucleases might be involved in spDSB generation.

In order to examine the roles of EndoG and CAD in caspase-mediated spDSB formation, we used the CRISPR technology to generate MDA-MB-231 cells with individual CAD or EndoG knockout or combined EndoG/CAD double-knockout (EndoG/CAD DKO) (Figure 2F). These cells were then evaluated for their spDSB levels by evaluating their γH2AX and 53BP1-mCherry foci levels (Figure 2G). Our results showed that both EndoG and CAD knockouts resulted in reduction of spDSB levels in host cells. In addition, EndoG/CAD DKO cells showed more reduction in spDSB levels than either knockout alone (Figure 2G). These results therefore established EndoG and CAD as key downstream effectors of caspases for generating spDSBs in tumor cells.

### Effects of spDSB formation on growth and tumorigenic abilities of cancer cells

Our data in Figure 2 and Supplementary information, Figure S2 clearly indicate that many tumor cells possess robust levels of spDSBs induced by cytochrome c leakage and sublethal activation of apoptotic caspase and nuclease. One important question is the cause of spontaneous cytochrome c leakage in cancer cells. Previously, it has been demonstrated JNK1 and JNK2 proteins are responsible for cytochrome c leakage in stress-induced apoptosis pathway^30^. We therefore examined the expression and activities of JNK1 and JNK2 in a panel of normal and cancer cells. Our results indicate that cancer cells appeared to possess higher levels of phosphorylated JNK1/JNK2 (Supplementary information, Figure S3A), indicating constitutive activation. When JNK1 and JNK2 were depleted in MDA-MB-231 cells, extra-mitochondrial cytosolic cytochrome c levels appeared to be reduced significantly (Supplementary information, Figure S3B), indicating that JNK1 and JNK2 are responsible for cytochrome c leakage as reported in apoptotic cells^30^. Furthermore, knockdown of JNK1/JNK2 also caused significant reduction in γH2AX foci formation (Supplementary information, Figure S3C; see Supplementary information, Table S4 for details on JNK1 and 2 targeting shRNA). These data thus support JNK1/JNK2 as upstream factors in promoting sublethal cytochrome c leakage and spDSB formation in cancer cells.

**Figure 3:**
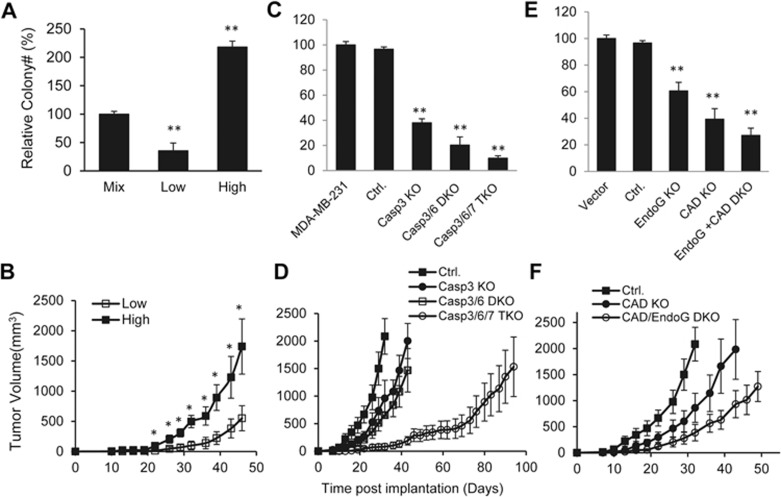
Effects of spDSBs on the tumorigenic abilities of cancer cells. **(A)** Relative numbers of soft agar colonies formed by unsorted (Mix) and sorted MDA-MB-231 cells with low and high levels of 53BP1-mCherry reporter activities. Error bars represent SEM (*n* = 3). Mix vs other group: ^**^P < 0.001; Student's *t*-test. **(B)** Tumor formation abilities in nude mice of MDA-MB-231 cells with low and high levels of 53BP1-mCherry reporter activities. Cells with higher level of 53BP1-mCherry reporter expression formed tumor at a significantly faster rate than those with lower reporter levels 22 days after the inoculation. Error bars represent SEM (*n* = 6); ^*^P < 0.05; Student's *t*-test. **(C)** Relative numbers of soft agar colonies formed by Casp3KO, Casp3/6DKO, and Casp3/6/7 TKO MDA-MB-231 cells. Ctrl., vector control. Error bars represent SEM (*n* = 3). Ctrl. vs other group: ^**^P < 0.001. **(D)** Tumor formation abilities of vector-transfected (Ctrl.), Casp3 KO, Casp3/6 DKO, and Casp3/6/7 TKO MDA-MB-231 cells injected subcutaneously (2 × 10^5^ per injection) into nude mice. Comparing the times for the tumors to reach 1 000 mm^3^ in volume, Casp3/6/7 TKO group took significantly longer than Ctrl., Casp3 KO, or Casp3/6 DKO groups (*P* < 0.001; *n* = 6; Student's *t*-test). Error bars represent SEM. **(E)** Relative numbers of soft agar colonies formed by EndoG KO, CAD KO, and EndoG/CAD DKO MDA-MB-231 cells. Error bars represent SEM (*n* = 3). Ctrl. vs other group: ^**^P < 0.001. **(F)** Tumorigenic abilities of vector-transfected and CAD KO and EndoG/CAD DKO MDA-MB-231 cells in nude mice. Comparing the times for the tumors to reach 1 000 mm^3^, both CAD KO (*P* = 0.0227) and EndoG/CAD DKO (*P* = 0.0026) groups took significantly longer times than the control group (Student's *t*-test; *n* = 6). The error bars represent SEM.

Currently, it is widely accepted that DSBs can cause genetic instability and cell cycle arrest. Therefore, it is reasonable to posit that spDSBs in tumor cells may slow down tumor cell growth by causing cell cycle arrest. However, cells with higher levels of spDSBs grew at rates similar to those of the control cells with doubling times of 31 and 32 h, respectively. Even more surprising is the fact that cells with higher level of spDSBs showed significantly enhanced clonogenic abilities in soft agar (Figure 3A and Supplementary information, Figure S3D), which evaluates anchorage-independent growth, a long-established assay for tumorigenicity^31^. In further experiments, MDA-MB-231 and HT29 cells transduced with the 53BP1-mCherry reporter were sorted by flow cytometry according to different levels of spDSBs (see Supplementary information, Figure S1F for a 53BP1-mCherry-based flow cytometry profile). When the sorted cells were evaluated for soft agar growth, cells with higher levels of spDSBs showed a progressively enhanced capacity to grow in soft agar (Supplementary information, Figure S3E and S3F). The only exceptions are cells with the highest levels of spDSBs (R6), which exhibited a reduced level of soft agar growth in both MDA-MB-231 (Supplementary information, Figure S3E) and HT29 (Supplementary information, Figure S3F) cells; however, these cells had a higher growth rate than those with the lowest level of spDSBs (R1 and R2). Consistent with their roles in promoting cytochrome c leakage and spDSB formation, combined JNK1/2 depletion also significantly reduced soft agar colony formation (Supplementary information, Figure S3G).

When MDA-MB-231 cells with differing spDSB levels were evaluated for their abilities to grow in nude mice, those with higher spDSB levels (from the R5 gate) formed tumor at a significantly faster rate than those with lower spDSB levels (from the R1 gate) (Figure 3B).

Because we have established that non-lethal activation of caspase-3, -6, and -7 plays major roles in the induction of spDSBs, we evaluated MDA-MB-231 cells with single, double, or triple knockout of the three caspases for their abilities to grow in soft agar. Compared with control cells, Casp3 KO, Casp3/6 DKO, or Casp3/6/7 TKO cells showed reduced ability to grow in soft agar (Figure 3C). When these cells were injected into nude mice, they also grew significantly slower (Figure 3D). It is interesting to note that cells with triple caspase knockout showed a greatly reduced growth rate when compared with vector control cells (Figure 3D). Consistently, MCF7-Casp3 cells exhibited enhanced ability to grow in soft agar (Supplementary information, Figure S3H) and to form tumors in nude mice (Supplementary information, Figure S3I) compared with the parental MCF7 cells. To further study the roles of mitochondrial membrane permeabilization on tumorigenicity of cancer cells, we carried out shRNA-mediated knockdown of BAX and BAK (Supplementary information, Figure S3J, left panels), two factors known to promote cytochrome c leakage^32^. BAX/BAK double-knockdown cells formed tumors in mice at a significantly slower rate (Supplementary information, Figure S3J). The importance of mitochondrial membrane permeabilization was further confirmed by use of MDA-MB-231 cells with cytochrome c knockout. These cells formed tumors in mice at a significantly slower rate than vector control cells (Supplementary information, Figure S3K), indicating an important role of cytochrome c in maintaining the tumorigenicity of cancer cells. These results, together with those shown in Figure 2, firmly established a causative role for executioner caspases in both spDSB induction and sustaining the tumorigenic abilities of the host cells.

To obtain direct evidence that spDSBs enhance the tumorigenic abilities of host cancer cells, we evaluated MDA-MB-231 cells with EndoG and/or CAD knockout. We have shown that these two apoptotic nucleases were directly responsible for the formation of a significant portion of the spDSBs (Figure 2F and 2G). Both EndoG and CAD knockouts significantly reduced the abilities of MDA-MB-231 cells to form colonies in soft agar (Figure 3E). EndoG/CAD DKO cells showed the weakest ability to grow in soft agar. When we injected these cells into nude mice, EndoG/CAD DKO cells showed significantly reduced rate of growth when compared with either the vector control cells or CAD-knockout cells (Figure 3F). These experiments therefore provide direct evidence that spDSBs induced by apoptotic nucleases EndoG and CAD play important roles in maintaining the tumorigenic abilities of the tumor cells.

### The effects of artificially induced DSBs on tumorigenicity of cancer cells

**Figure 4:**
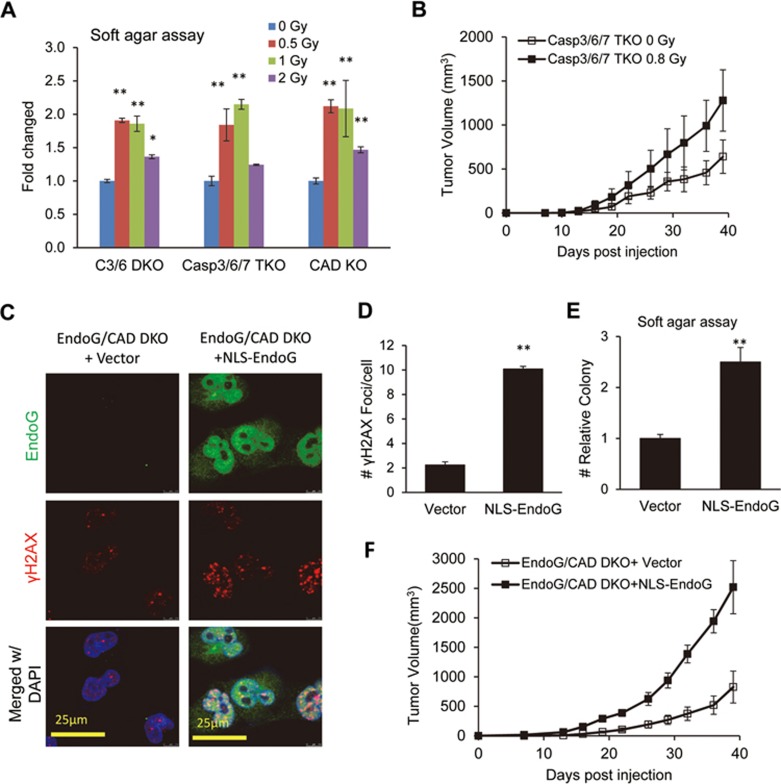
The effects of artificially induced DSBs on tumor cell growth *in vitro* and *in vivo*. **(A)** Relative soft agar growth in low-dose X-ray-irradiated MDA-MB-231 cells with Casp3/6 DKO, Casp3/6/7 TKO, and CAD KO. Error bars represent SEM (*n* = 3). 0 Gy vs other groups: ^*^P < 0.05; ^**^P < 0.001. **(B)** Xenograft tumor formation ability of Casp3/6/7 TKO MDA-MB-231 cells (6 × 10^5^ cells per tumor) was significantly enhanced (*P* < 0.05) by exposure to a low-dose of X-rays (0.8 Gy) 22 days after inoculation. Error bars represent SEM (*n* = 6; Student's *t*-test). **(C)** Microscopic images of EndoG (green) and γH2AX foci staining in non-transduced and *NLS-EndoG* transduced EndoG/CAD DKO MDA-MB-231 cells. Notice the significant increase in γH2AX foci in the transduced cells. **(D)** Quantitative analysis of the number of γH2AX foci in *NLS-EndoG*-transduced and non-transduced EndoG/CAD DKO MDA-MB-231 cells. Error bars represent SEM (*n* = 3). ^**^P < 0.001; Student's *t*-test. **(E)** Relative soft agar colony formation of *NLS-EndoG*-transduced and non-transduced EndoG/CAD DKO MDA-MB-231 cells. Error bars represent SEM (*n* = 3). ^**^P < 0.001; Student's *t*-test. **(F)** Enhanced tumor formation abilities of *NLS-EndoG*-transduced EndoG/CAD DKO MDA-MB-231 cells when compared with non-transduced cells. The differences between the *NLS-EndoG*-transduced group vs the vector-transduced group were significant (*P* < 0.05) 13 days after initial tumor injection (2 × 10^5^ cell per tumor). Error bars represent SEM (*n* = 6 and 8 for vector-transduced group and *NLS-EndoG*-transduced group, respectively). Student's *t*-test was used.

Our results so far have clearly shown that spDSBs in tumor cells drive tumorigenicity. We next asked whether artificially induced DSBs are capable of doing the same. We attempted to artificially induce DSBs by use of two approaches:

In the first approach, we used X-rays, which are known to induce DSBs efficiently. We chose to use MDA-MB-231 cells with Casp3/6 DKO, Casp3/6/7 TKO, or CAD knockout because these cells have low background levels of spDSBs. The cells were irradiated with low-level X-ray doses ranging from 0.5 to 2 Gy that kept most of the irradiated cells alive with the exception of 2 Gy (Supplementary information, Figure S4A). Despite a small effect on cell death, low-dose radiation (0.8 Gy) caused a transient increase in γH2X foci numbers that lasted for 4-24 h (Supplementary information, Figure S4B). At the molecular level, low-dose radiation also induced the activation of cell cycle check point proteins Chk1 and Chk2 for up to 48 h (Supplementary information, Figure S4C). Despite DSB induction and cell cycle check point activation, low-dose X-ray irradiation boosted the soft agar colony formation of the tumor cells (Figure 4A), consistent with the hypothesis that spDSBs are sufficient in promoting tumorigenicity. At 2 Gy, the colony formation was reduced but still above sham-irradiated cells. The reduction in colony formation at 2 Gy probably reflected increased radiation-induced cell death at that dose.

To evaluate the influence of radiation-induced DSBs on tumor formation *in vivo*, we injected sham-irradiated and X-ray-irradiated Casp3/6/7 TKO cells into nude mice. Instead of slowing down tumor formation, low-dose X-ray exposure significantly boosted growth of the Casp3/6/7 TKO cells (Figure 4B).

In the second approach, we engineered a truncated EndoG gene in which we replaced the mitochondria localization signal peptide (aa1-48) with a nuclear localization signal (NLS) from the SV40 large T antigen so that EndoG will go directly into the nucleus instead of the mitochondria (Supplementary information, Figure S4D). After transduction of the engineered NLS-EndoG into EndoG/CAD DKO cells, we found that the transduced cells showed significantly higher levels of γΗ2ΑΧ foci (Figure 4C and 4D), confirming that transduced NLS-EndoG induced a significantly higher levels of DSBs. At the molecular level, NLS-EndoG transduction caused increased phosphorylation of Chk2. However, increased γH2AX foci induction and Chk2 activation (Supplementary information, Figure S4E) appeared to have minimal effect on the growth rate of MDA-MB-231 cells (Supplementary information, Figure S4F). We subsequently evaluated the soft agar growth capability of the transduced cells and found that NLS-EndoG-transduced cells formed soft agar colonies at a much higher level than their parental cells (Figure 4E).

We also evaluated the effect of NLS-EndoG transduction on the tumorigenic abilities of EndoG/CAD DKO cells in nude mice. When the NLS-EndoG-transduced cells were injected subcutaneously into nude mice, they formed xenograft tumors at a much faster rate (Figure 4F), consistent with results obtained in the soft agar assay.

Therefore, in both of our approaches to artificially induce DSBs in cells with relatively low levels of spDSBs, we were able to enhance the tumorigenic abilities of the host cells significantly. These experiments thus provide further evidence for a pivotal role of spDSBs in driving tumorigenicity.

### Activation of ATM/ATR by spDSBs and its role in driving tumor growth

**Figure 5:**
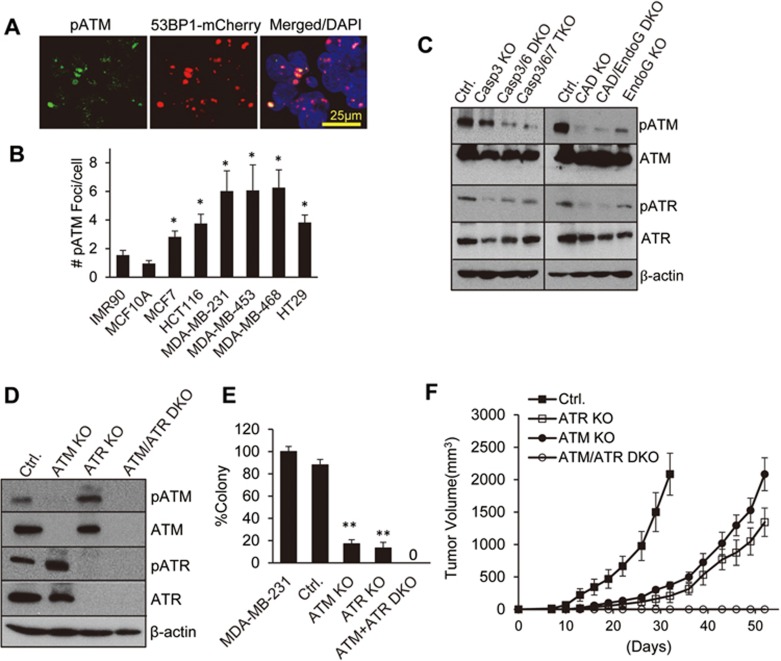
Roles of ATM/ATR in maintaining the tumorigenicity of cancer cells. **(A)** Co-localization of pATM foci and 53BP1foci in 53BP1-mCherry-transduced MDA-MB-231 cells. **(B)** Quantitative analysis of pATM foci in various transformed and non-transformed cell lines. Error bars represent SEM (*n* = 3). IMR90 vs other groups: ^*^P < 0.05. **(C)** Western blot analysis of pATM and pATR expression in caspase-3, -6, -7 (left panels), CAD, and EndoG knockout MDA-MB-231 cells. **(D)** Western blot analysis of ATM and ATR expression in CRISPR/Cas9-mediated ATM KO, ATR KO, and ATM/ATR DKO MDA-MB-231 cells. **(E)** Relative soft agar colony formation in ATM KO, ATR KO, and ATM/ATR DKO MDA-MB-231 cells. Error bars represent SEM (*n* = 3). Ctrl. vs other group: ^**^P < 0.001. **(F)** Tumorigenic abilities of vector-transduced, ATM KO, ATR KO, and ATM/ATR DKO MDA-MB-231 cells in nude mice. ATM/ATR DKO cells cannot form any tumors. Comparing the times to reach 1 000 mm^3^ in tumor volumes, both ATM KO (*P* < 0.001; Student's *t*-test), or ATR KO (*P* < 0.001; Student's *t*-test) groups took significantly longer when compared with Ctrl. group. Error bars represent SEM (*n* = 6).

We next tried to elucidate the mechanisms of how spDSBs enhance tumorigenicity. To answer this question, we performed immunofluorescence analysis of phosphorylated ATM (pATM) in MDA-MB-231 cells. Activation of ATM through autophosphorylation is a key event in cellular DDR^6^. Our results showed clear presence of pATM foci in cells with a significant number of 53BP1-mCherry foci (Figure 5A). Importantly, most of the pATM foci overlap with 53BP1-mCherry foci, as expected from true DSB-related foci. These results were consistent with western blot analysis of pATM in cells with low or high levels of 53BP1-mCherry foci. Cells with higher level of 53BP1-mCherry foci possessed higher levels of pATM (Supplementary information, Figure S5A). A survey of a panel of non-transformed and transformed cells indicates that similar to 53BP1 and γH2AX foci, all tumor cells possessed higher numbers of pATM foci than non-transformed cells (Figure 5B). Interestingly, MCF7 cells, which do not express caspase-3, have the fewest number of pATM foci among the cancer cells examined.

We also carried out western blot to analyze phosphorylation of ATM and ATR, the latter of which is an ATM-related protein also involved in DDR and cell cycle check point control^33,34^. We observed a robust, constitutive ATM phosphorylation in parental MDA-MB-231 cells (Figure 5C). Interestingly, cells with caspase knockouts showed significantly reduced pATM levels with double- or triple-knockout cells showing greater pATM reduction than caspase-3 single knockout cells (Figure 5C, top panel). Furthermore, we showed that both CAD- and/or EndoG-knockout cells exhibited significantly reduced pATM levels with CAD-knockout or EndoG/CAD DKO cells showing greater reduction than EndoG-knockout cells. We also showed that ATR, which is usually associated with single-strand DNA and UV damage^35^, was activated in control MDA-MB-231 cells as demonstrated by its constitutive phosphorylation (Figure 5C, third panel from the top). Similar to ATM, pATR levels were reduced in both apoptotic caspase- and nuclease-knockout cells (Figure 5C). The western blot results demonstrating the relationship between caspases/apoptotic nucleases and ATM activation were further confirmed at the individual cell level showing clear co-staining of pATM and activated caspase-3 (Supplementary information, Figure S5B). In addition, the fraction of cells with pATM staining was significantly reduced in both Casp3/6/7 TKO cells and EndoG/CAD DKO cells (Supplementary information, Figure S5C and S5D).

In order to determine whether ATM and ATR activation is functionally relevant, we generated MDA-MB-231 cells with ATM, ATR, or ATM/ATR single or double-knockouts (Figure 5D). ATM- and ATR-knockout cells showed minimal cellular stress and proliferated at a similar rate as control cells (data not shown). However, they did show some morphological changes (Supplementary information, Figure S5E). The fact that we were able to generate ATR knockout was in itself remarkable. In previous studies, it was shown that ATR knockout caused cell death within a few cell divisions^36^. However, MDA-MB-231 cells with ATR knockout appeared to do fine, albeit with increased cellular death and morphological changes. This is perhaps a reflection of less stringency and more redundancy between the functions of ATM and ATR. Indeed, ATM/ATR double-knockout (ATM/ATR DKO) cells showed a significantly slower growth rate, increased cell death level, and eventual cellular senescence at the whole-population level after extended culturing. Interestingly, in ATR-knockout cells, pATM level increased when compared with control cells (Figure 5D, top panel). In ATM-knockout cells, pATR level increased significantly when compared with control cells (Figure 5D, third panel from the top). Therefore, ATM and ATR were mutually compensatory in terms of their activation when either one of them was missing.

The importance of ATM and ATR was demonstrated by the fact that both the single knockout (ATM or ATR) cells and ATM/ATR DKO cells showed greatly reduced ability to form soft agar colonies (Figure 5E). In fact, the double-knockout cells could not form any soft agar colonies.

In another experiment, we attempted to determine if the kinase activity of ATM is important for its role in maintaining tumorigenicity. MDA-MB-231-ATM KO cells were transduced with either wild-type ATM or a catalytically inactive kinase dead (KD) version of ATM gene^5^ (Supplementary information, Figure S5F). These cells were then evaluated for their ability to form soft agar colonies. Although re-expression of wild-type ATM completely restored colony-forming abilities of MDA-MB-231 cells, the KD version did not (Supplementary information, Figure S5G), suggesting a critical role for the kinase activities in tumorigenicity.

We further examined the abilities of ATM/ATR-knockout cells to form tumors in nude mice. Both ATM- and ATR-knockout cells showed significantly reduced ability to grow as tumors when compared with control cells (Figure 5F). Consistently, ATM/ATR DKO cells could not form tumors at all. The importance of ATM in supporting invasive tumor cell growth was also shown in MCF7 (Supplementary information, Figure S5H), HT29 (Supplementary information, Figure S5I), and HCT116 (Supplementary information, Figure S5J) cancer cells. In these three cell lines, soft agar growth was significantly reduced when ATM gene was knocked out. Our results therefore demonstrate that both ATM and ATR play critical roles in sustaining tumorigenicity. This is surprising given that both ATM^10,11,12,13^ and ATR^36^ had tumor-suppressive activities in earlier studies.

### SpDSB-induced activation of NF-κB and STAT3

**Figure 6:**
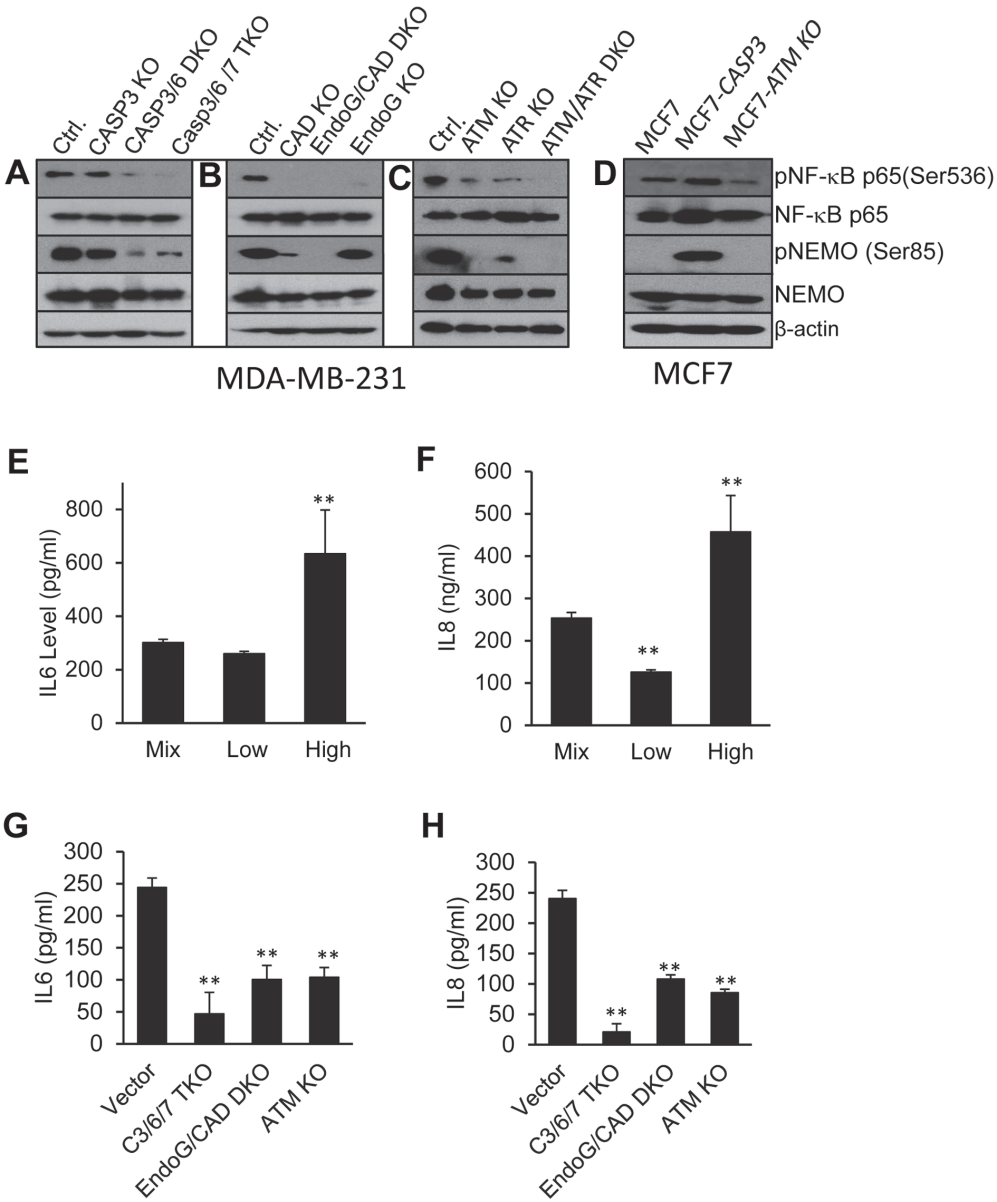
SpDSB-induced activation of the transcription factor NF-κB. **(A)** Western blot analysis of phosphorylated NF-κB p65 and NEMO expression in caspase-3,-6,-7-knockout MDA-MB-231 cells. **(B)** Western blot analysis of phosphorylated NF-κB p65 and NEMO expression in CAD- and/or EndoG-knockout MDA-MB-231 cells. **(C)** Western blot analysis of phosphorylated NF-κB p65 and NEMO expression in ATM- and/or ATR-knockout MDA-MB-231 cells. **(D)** Western blot analysis of phosphorylated NF-κB p65 and NEMO expression in parental MCF7, MCF7-Casp3, and MCF7-ATM KO cells. **(E**, **F)** IL6 **(E)** and IL8 **(F)** levels in the supernatants of low and high 53BP1-mCherry-expressing MDA-MB-231 cells as detected by ELISA. Error bars represent SEM (*n* = 3; ^**^P < 0.001; Student's *t*-test). **(G**, **H)** IL6 **(G)** and IL8 **(H)** levels in supernatants of vector control and various knockout MDA-MB-231 cells as measured by ELISA. Error bars represent SEM (*n* = 3). Vector vs other group: ^**^P < 0.001; Student's *t*-test.

Now that we have established spDSB-activated ATM/ATR to be important for sustaining tumor growth, the next logical question is which downstream factors of ATM are responsible for supporting tumorigenicity. Previously, DSB-activated ATM has been shown to activate NF-κB through the phosphorylation of NEMO^37^, a positive modulator of NF-κB that exports to the cytoplasm from the nucleus upon ATM-mediated phosphorylation. In addition, DSB-mediated ATM activation has also been shown to activate NF-κB p65/RelA in the cytoplasm^38^. Indeed, NEMO and p65 were phosphorylated in control MDA-MB-231 cells (Figure 6A and 6B), consistent with NF-κB being constitutively activated. In both caspase-knockout (especially Casp3/6 DKO and Casp3/6/7 TKO) (Figure 6A) and EndoG/CAD DKO cells (Figure 6B), phosphorylation of p65/RelA and NEMO was significantly reduced, suggesting reduced NF-κB activation. These results were remarkable given that apoptotic nucleases CAD and EndoG have never been shown to be associated with NF-κB activation. The clear causal relationships between caspases/EndoG/CAD and NF-κB activation thus provide the strongest evidence for the role of spDSBs in driving tumorigenicity.

Consistently, in ATM/ATR individual or double-knockout cells, phosphorylation for both NEMO and p65/RelA was significantly reduced (Figure 6C). These results therefore were consistent with NF-κB being constitutively activated in tumor cells by activated ATM and ATR, which were themselves shown to be activated by the presence of spDSBs earlier (Figure 5).

We further confirmed the caspase-ATM-NF-κB relationship in MCF7 cells (Figure 6D). When MCF7 cells were transduced with the caspase-3 gene, phosphorylation of p65/RelA and NEMO was significantly increased, suggesting enhanced NF-κB activation. On the other hand, phosphorylation of p65/RelA and NEMO was significantly reduced by knockout of ATM in MCF7 cells.

The relationship between caspases/EndoG/CAD/ATM and NF-κB was further confirmed at the individual cell level through immunofluorescence staining, which showed reduced pNEMO staining in MDA-MB-231 cells with Casp3/6/7 TKO or EndoG/CAD DKO, indicative of constitutive NF-κB activation (Supplementary information, Figure S6A and S6B).

Two of the most important downstream factors of NF-κB shown to be important for tumor growth are pro-inflammatory, pro-angiogenic factors IL6 and IL8^39,40,41^. We measured the levels of these two cytokines in the supernatants of MDA-MB-231 cells with high or low 53BP1-mCherry reporter activities. Cells with higher levels of reporter activity had higher levels of cytokines in their supernatants (Figure 6E and 6F). We also measured the levels of these two cytokines in the supernatants of several knockout cell lines. Secretion of both IL6 and IL8 was significantly reduced in Casp3/6/7 TKO, EndoG/CAD DKO, and ATM-knockout cells (Figure 6G and 6H), confirming the NF-κB analysis (Figure 6A-6D).

We next analyzed the activation status of STAT3, a transcription factor important for both tumor growth^42,43^ and maintenance of stemness in embryonic stem cells^44,45^. STAT3 has been shown to act downstream of NF-κB and ATM. IL6 is known to activate STAT3 through the Jak kinase^46^. Western blot analysis indicates that in Casp3/6/7, CAD, and EndoG-knockout cells, the level of phosphorylated STAT3 (pSTAT3), which is the activated form of STAT3, was significantly reduced (Supplementary information, Figure S6C and S6D). In addition, pSTAT3 level was also reduced in ATM-, ATR-, and ATM/ATR-knockout cells (Supplementary information, Figure S6E), consistent with ATM/ATR acting upstream of STAT3. Furthermore, we confirmed the results in MCF7 cells. The pSTAT3 (Y705) level was increased in Casp3-overexpressing cells (MCF7-Casp3). In contrast, it was decreased in ATM-knockout MCF-7 cells (Supplementary information, Figure S6F).

To establish the functional importance of STAT3 as a downstream effector of active ATM in maintaining the tumorigenicity of cancer cells, we transduced a constitutively active (STAT3C) or a dominant-negative (STAT3DN) version of STAT3^47^ into ATM-knockout MDA-MB-231 breast cancer cells and D456MG patient-derived glioma cells. The goal was to determine whether the deficiency in tumorigenicity caused by ATM knockout could be rescued by STAT3 activation. Our soft agar colony formation experiments using these cells demonstrated that constitutively active STAT3C, but not STAT3DN, could rescue tumorigenicity in both MDA-MB-231-ATM KO and D456MG-ATM KO cells (Supplementary information, Figure S6G and S6H). These results provide strong evidence that STAT3 is indeed a key downstream effector of ATM in maintaining tumorigenicity.

### The importance of spDSBs in maintaining the stemness of patient-derived glioma cells

**Figure 7:**
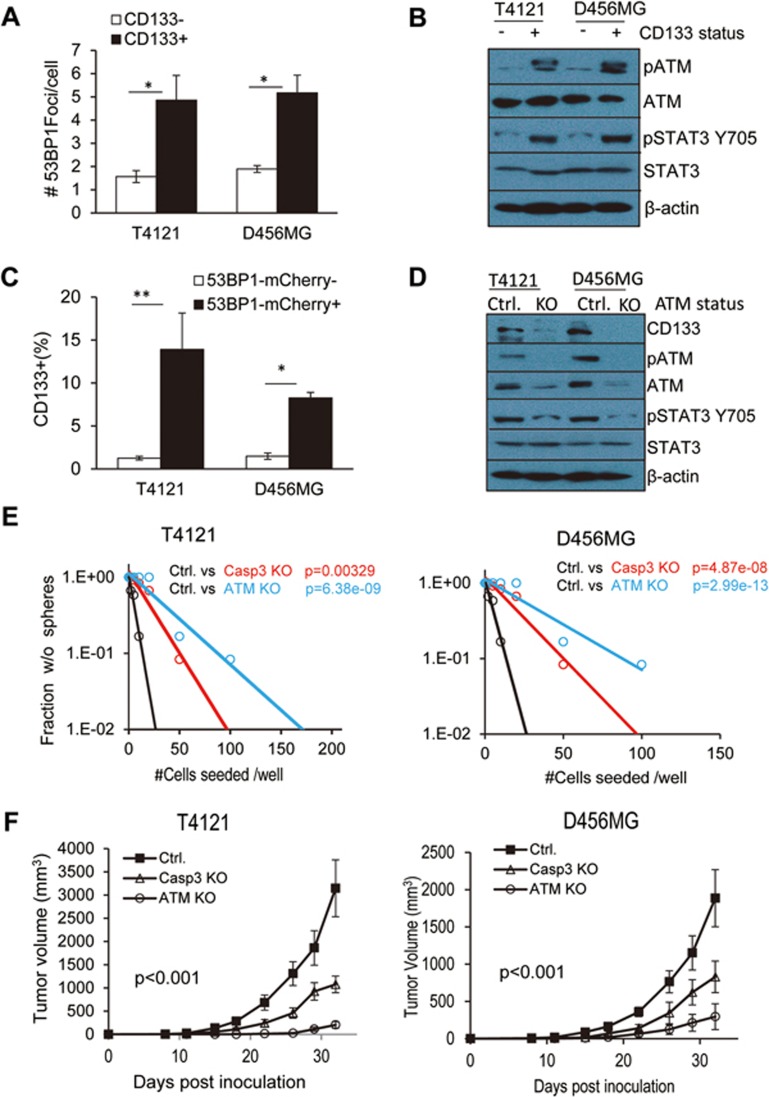
The association of spDSB-induced DNA damage response with stemness and tumor-initiating abilities of patient-derived glioma cells. **(A)** 53BP1 foci numbers in sorted CD133^−^ and CD133^+^ patient-derived glioma cells (T4121 and D456MG). Error bars represent SEM (*n* = 3); ^*^P < 0.05; Student's *t*-test. **(B)** Western blot analysis of pATM and pSTAT3 levels in CD133^−^ and CD133^+^ glioma cells (T4121 and D456MG). **(C)** Fraction of cells with positive CD133 expression in 53BP1-mCherry high (+) and low (−) glioma cells (T4121 and D456MG). Error bars represent SEM (*n* = 3); ^*^P < 0.05; ^**^P < 0.001; Student's *t*-test. See Supplementary information, Figure S7C for criteria for 53BP1-mCherry-high or -low cells. **(D)** Western blot analysis of CD133 and pSTAT3 expression in glioma cells (T4121 and D456MG) with or without *ATM* gene knockouts. **(E)** Limited dilution analysis of tumor sphere formation abilities of patient-derived T4121 and D456MG glioma cells with genetic knockouts of Casp3 and ATM. *P*-values were calculated from χ^2^-test. **(F)** Tumor growth of T4121 and D456MG glioma cells with vector control, Casp3 KO or ATM KO. About 1 × 10^6^ cells were injected subcutaneously in severe combined immunodeficiency (SCID) mice (*n* = 6) and the rate of tumor formation in mice was followed for 32 days. The differences between the groups are statistically significant for both glioma tumor cells (*P* < 0.001; two-way ANOVA test).

Because of the well-established role of STAT3 in cancer stem cells and embryonic stem cells, we decided to examine whether our newly discovered spDSBs play any roles in glioma stem cells. By use of established protocols^48^, we sorted two patient-derived glioma lines into CD133^+^ and CD133^−^ fractions (see Supplementary information, Figure S7A for gating criteria). CD133 is a well-established cell surface marker for glioma stem cells^49,50^. Consistent with published reports^49,50^, CD133^+^ cells formed much bigger tumor spheres, indicating their cancer stem cell status (Supplementary information, Figure S7B). We then determined the presence of DNA DSB foci by quantifying the number of 53BP1 foci in the sorted cells. Interestingly, in both human glioma lines, the CD133^+^ cells had significantly more 53BP1-mCherry foci (Figure 7A), indicating the presence of more spDSBs in glioma stem cells. Western blot analysis confirmed the 53BP1-mCherry foci results. Cells with high CD133 expression levels showed increased levels of pATM and pSTAT3 (pSTAT3-Y705) (Figure 7B). Therefore, our experiments clearly showed that glioma stem cells have more spDSBs.

In a subsequent experiment, we attempted to determine whether cells with higher amounts of spDSBs have higher fractions of glioma stem cells. We transduced 53BP1-mCherry into T4121 and D456MG glioma cells. The cells were then sorted into 53BP1-mCherry-low and -high fractions (see Supplementary information, Figure S7C for gating criteria). The high and low factions of the cells were then analyzed for their expression of CD133 by flow cytometry. In both glioma cell lines, those with higher levels of spDSBs, as represented by higher levels of 53BP1-mCherry activities, had significantly higher levels of CD133 expression (Figure 7C).

In order to show a causal relationship between spDSB-induced ATM activation and stemness of the glioma cells, we used a multiplex CRISPR-sgRNA vector to target the ATM gene. Western blot analysis showed that we were able to significantly reduce ATM expression in both T4121 and D456MG cells (Figure 7D). Interestingly, compared with vector control-transduced cells, glioma cells with ATM knockout showed significantly less CD133 expression and reduced levels of activated STAT3. These results suggest that ATM activation is directly responsible for STAT3 activation and CD133 expression, two important markers for the stemness of glioma cells. Additional western blot analysis indicates that ATM knockout also caused significantly reduced expression of Oct4 and Nestin (Supplementary information, Figure S7D), two important markers of glioma stem cells. Though never reported for cancer stem cells, the importance of ATM for glioma stem cells is reminiscent of the requirement of ATM in the self-renewal of hematopoietic stem cells^51^.

To demonstrate the functional relevance of the spDSB-induced DDR pathway in the stemness of glioma cells, we examined tumorigenic abilities of the patient-derived glioma cells with caspase-3 or ATM knockout. We carried out three different assays. In the first assay, we evaluated the stemness of the knockout cells by using the limited dilution assay to determine the cells' abilities to form tumor spheres in suspension culture. Our results indicate that glioma cells with caspase-3 or ATM knockout had significantly reduced abilities to form tumor spheres than control in both T4121 and D456MG glioma cells (Figure 7E). In the second assay, we showed that in both T4121 and D456MG glioma cells, ATM or caspase-3 knockout reduced the ability of the glioma cells to grow in soft agar (Supplementary information, Figure S7E). In the third assay, we evaluated the abilities of the caspase-3 or ATM-knockout glioma cells to form tumors in mice. Our results indicate that ATM and caspase-3 knockout caused significant attenuation in the abilities of glioma cells to grow in immunodeficient mice (Figure 7F).

To establish the functional relevance of STAT3 as a downstream effector of ATM in maintaining the stemness of glioma stem cells, we transduced D456MG-ATM KO cells with a constitutively active STAT3 (STAT3C) or a dominant-negative STAT3 (STAT3DN). We then examined the transduced cells for their CD133 expression by western blot analysis. Our results indicate that CD133 expression, which was significantly attenuated in ATM KO cells, was restored by STAT3C but not STAT3DN expression (Supplementary information, Figure S7F). In addition, tumor sphere-forming abilities of the D456MG-ATM KO cells were also restored by STAT3C, but not by STAT3DN or vector expression (Supplementary information, Figure S7G).

Taken together, the above results firmly establish a close, inter-woven relationship between spDSBs, activated ATM, STAT3, and CD133 expression, underscoring the importance of spDSBs in driving tumorigenicity and stemness of glioma cells.

### The relevance of spDSB-induced DDR in human cancers

**Figure 8:**
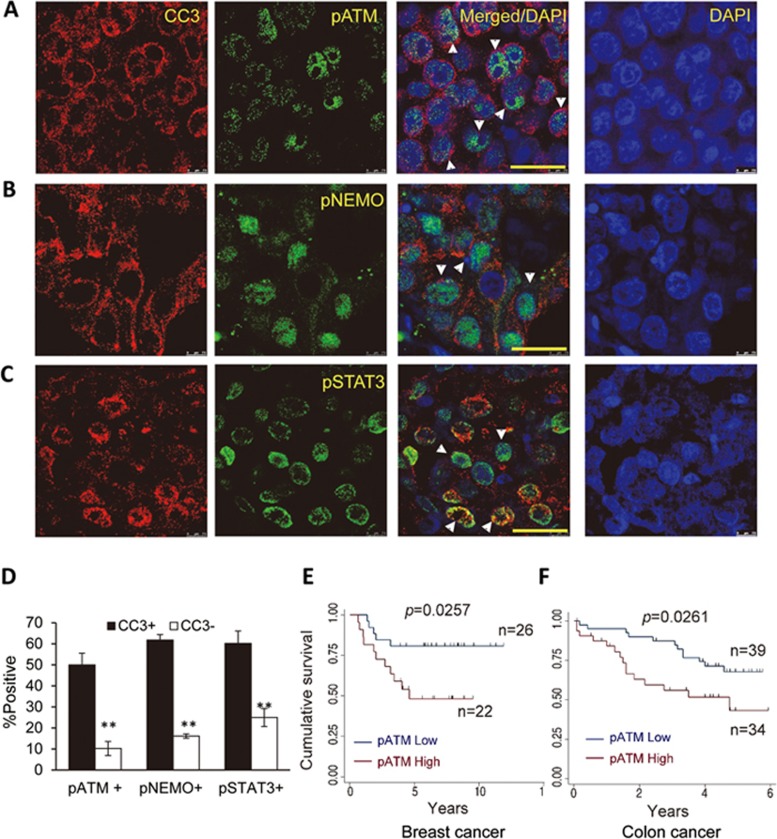
Immunofluorescence and immunohistochemistry analysis of factors involved in spDSB induction and DNA damage response in human tumor samples and their relevance in prognosis. **(A**-**C)** Confocal microscope imaging of immunofluorescence co-staining of cleaved caspase3 (CC3) with pATM **(A)**, pNEMO **(B)**, and pSTAT3-Y705 **(C)** in human breast cancer tissues. White arrowheads in the merged images indicate cells with double positive staining. Scale bar, 20 μm. **(D)** Quantitative estimate of the fraction of cells positive for pATM, pNEMO, and pSTAT3-Y705 among CC3^+^ or CC3^−^ cells. Error bars represent SEM (*n* = 3). ^**^P < 0.001; Student's *t*-test. **(E)** Kaplan-Meier survival analysis of a cohort of breast cancer patients. Plotted is patient survival rate for those with high or low levels of pATM in their pre-treatment tumor samples. *P* = 0.0257; *n* = 48; log-rank test. **(F)** Kaplan-Meier survival analysis of a cohort of colon cancer patients. Plotted is patient survival rate for those with high or low levels of pATM in their pre-treatment tumor samples. *P* = 0.0261; *n* = 73; log-rank test.

In order to validate the relevance of our discoveries on spDSBs in human cancers, we conducted immunofluorescence staining analysis of tumor tissue samples from pre-treatment human breast cancer patients. Our results demonstrate clear co-staining of activated caspase-3 (or cleaved caspase-3, CC3) with pATM (Figure 8A), activated NF-κB (as represented by pNEMO) (Figure 8B) and pSTAT3 (Figure 8C) in intact, non-apoptotic cells. In addition, we also observed co-staining of nuclear EndoG with pATM (Supplementary information, Figure S8A). Quantitative analyses indicate that over 50% of tumor cells that stained positive for activated caspase-3 (CC3^+^) also stained positive for pATM, pNEMO, and pSTAT3, significantly more than those in CC3^−^ cells (Figure 8D) . Similarly, over 70% of cells that stained positive for nuclear EndoG (nEndoG^+^) also stained positive for pATM, whereas < 20% nEndoG^−^ cells stained positive for pATM (Supplementary information, Figure S8B). These results indicate clear presence of spDSBs and DDR in human breast cancer samples.

We also conducted immunohistochemistry analysis of pATM in two cohorts of tumor samples (breast cancer and colon cancer) obtained before chemo- or radiotherapy (see Supplementary information, Tables S5 and S6 for details on patient characteristics). In some of the patient-derived tumor samples from both cohorts, there was widespread activation of ATM as demonstrated by strong nuclear pATM staining (Supplementary information, Figure S8C, lower panels). On the other hand, there were also samples from both cohorts showing only low-level staining of pATM (Supplementary information, Figure S8C, top panels). Strikingly, in breast cancer (Figure 8E) as well as in colon cancer (Figure 8F) patient cohorts, patients whose tumors exhibited high levels of pATM staining had significantly shorter survival when compared with patients whose tumors showed low levels of pATM staining. Consistently, in these two cohorts, high levels of activated caspase-3 (CC3) correlated with poor patient survival (Supplementary information, Figure S8D and S8E).

Our results in the human tumor tissues are thus consistent with our earlier observations that spDSB-induced ATM activation leads to a more aggressive tumor phenotype. More importantly, these results demonstrate the relevance of spDSB-driven tumorigenic phenotype in human cancers.

## Discussion

**Figure 9:**
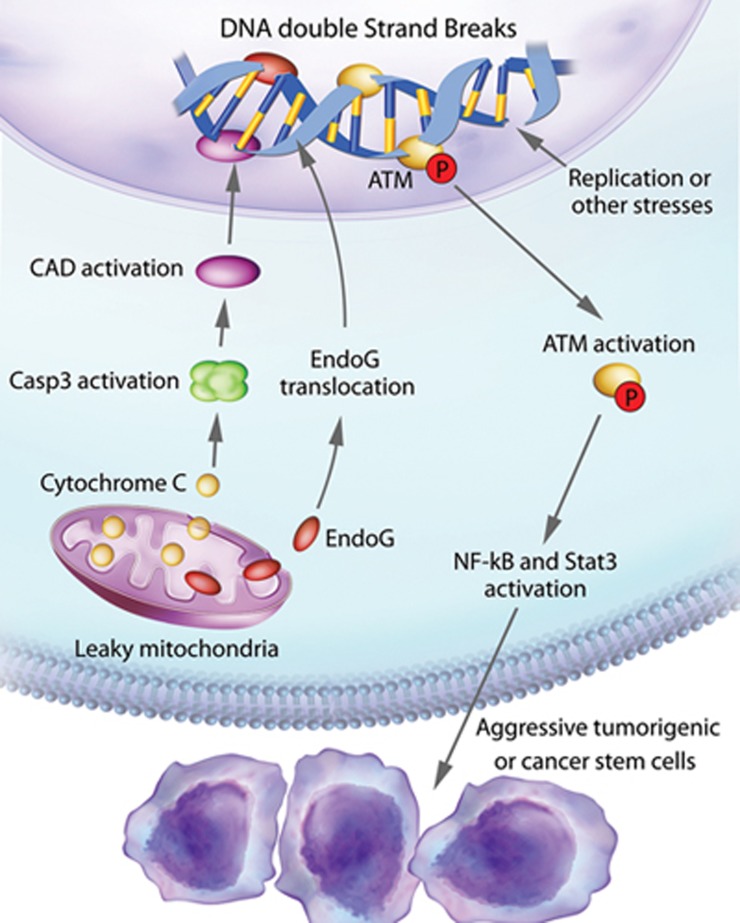
An illustration of induction of spontaneous DNA DSBs and their roles in maintaining the stemness and tumorigenicity of cancer cells. Our study shows that mitochondrial permeability changes in cancer cells allow spontaneous, sublethal activation of the apoptotic cascade that includes the cytoplasmic leakage of cytochrome c, caspase-3 activation, CAD activation, and EndoG nuclear translocation. Both CAD and EndoG are involved in causing spontaneous DNA DSBs in addition to those caused by replication stress or other stresses such as ROS. The presence of SpDSBs activates the DNA damage response (DDR). A key factor in DDR is ATM, which is persistently activated and thus remains in its phosphorylated form due to the constant presence of spDSBs. Phosphorylated ATM activates NF-κB and Stat3, two factors well known to the maintenance of tumorigenicity and stemness of cancer cells.

DNA DSBs are usually thought of as key source of chromosome aneuploidy that is a major feature of malignant cancer cells. They can also cause insertions and deletions in oncogenes and tumor suppressor genes that lead to oncogenic transformation. The two most surprising aspects of the present study are (1) the existence of spontaneously arising DNA DSBs caused by sublethal activation of apoptotic caspases and nucleases, which expands intrinsic sources of DNA DSB induction beyond those of DNA replication stress and reactivation oxygen species. The importance of our newly revealed mechanism is reflected by the fact that close to 50% of spontaneously arising DSBs are caused by sublethal caspase activation; (2) that spDSBs by themselves, irrespective of their effects on chromosomal aneuploidy and gene mutations, play a key role in driving tumorigenicity and stemness of cancer cells through persistent activation of ATM and DDR (Figure 9). To our knowledge, these findings have not been reported before.

The fact that cytochrome c leakage and ensuing apoptotic caspase, and nuclease activation cause persistent DBSs in healthy cancer cells is surprising given that these enzymes are generally associated with cells under stress and on the verge of death. However, our findings of spontaneous cytochrome c leakage, caspase activation, and resulting DNA damage being common in cancer cells but rare in non-transformed cells point to spontaneous, non-lethal mitochondrial leakage as a key distinguishing factor between normal and malignant cells. Although not described previously in unstressed cancer cells, there is recent literature on the roles of limited mitochondrial leakage in causing genetic instability and oncogenic transformation in stress-exposed cells^24,25,26^. Our findings are also consistent with previous reports that other forms of mitochondria-initiated signaling such as ROS generation are important in the maintenance of malignant cells^52^.

Although counter-intuitive at first sight, our results are consistent with and may provide further insights into some important previous observations made in cancer stem cells. An earlier study discovered that glioma stem cells had preferential activation of DNA repair proteins and thus were better equipped to deal with radiation-induced DNA damage^48^. However, it is unknown how the glioma stem cells possessed higher levels of activated DNA repair proteins. Our findings of spontaneously arising DSBs driving ATM activation provide a good explanation for the elevated DDR inherent in the glioma stem cells. Most interestingly, we showed that glioma cell stemness appeared to depend on spDSB-induced ATM activation. In the absence of caspase-3 and ATM, the expression of stem cell marker was greatly reduced. The dependence of glioma stemness on activated caspase-3 and ATM is consistent with requirement of ATM for the self-renewal of hematopoietic stem cells^51^. It is also consistent with our finding that human breast and colon tumors with higher levels of activated ATM have poor prognosis (Figure 8): a higher level of activated ATM may indicate higher numbers of malignant cancer stem cells. It will be interesting to see whether this association between cancer cell stemness and spDSB-induced ATM activation also occurs in more tumor types. Although presently unexplored, it is also likely that spDSB-driven stemness pathway similar to that observed in glioma stem cells may play a role in carcinogenesis. Such a pathway may facilitate carcinogenesis in combination with activated oncogenes. It will be important to examine such a possibility in future research.

One important implication for our discovery of spDSB-induced tumorigenicity and cancer cell stemness pathway is translational: it provides a strong, mechanism-based rationale for targeting the ATM/ATR pathway in cancer treatment. ATM inhibitors were initially developed as adjuvant agents for radiotherapy because of the perceived benefits in enhancing radiation sensitivity of cancer cells. Our discovery of a spDSB-induced tumorigenicity and cancer cell stemness pathway would provide a good rationale for the use of these agents as standalone inhibitors for the treatment of at least some cancers.

Taken together, the present study reveals the unexpected existence of spDSBs in cancer cells. As a result, the DDR proteins ATM/ATR are activated. Instead of causing cell cycle arrest or apoptosis, such activation serves as a key mechanism to maintain or enhance tumorigenicity and cancer cell stemness. The hitherto undescribed pathway provides exciting new insights into cancer biology and may have important implications in cancer treatment.

## Materials and Methods

### DNA damage assays

In order to evaluate DNA DSBs in tumor cells, we used a variety of assays that include γH2AX detection^20^, live imaging of 53BP1 foci through the use of a 53BP1-mCherry reporter^21^, and the comet assay^22^. See more details in the Supplementary information, Data S1.

### Evaluation of cytochrome c leakage

Cytochrome c leakage out of the mitochondria was evaluated by immunofluorescence staining and by western blot analysis of lysates fractionated into cytoplasmic and mitochondrial factions.

### CRISPR/Cas9-mediated gene knockout

Gene knockouts in various cancer cells were carried out according to published methods by use of vectors deposited by Dr Feng Zhang of MIT and the online tools developed by the same laboratory^53,54^.

### Animal experiments

All animal experiments were conducted in the Duke University vivarium following protocols approved by the Duke University Institutional Animal Use and Care Committee.

### Patient-derived tumor specimens

Analysis of levels of pATM and other molecular factors was conducted by use of the pre-treatment samples obtained from patients at the Shanghai General Hospital with approval of the Medical Ethics Committee of the hospital.

Please refer to Supplementary information, Data S1 for additional details on Materials and Methods.

## Author Contributions

XL and CL conceived of the study and designed the experiments. XL carried out most of the experiments. ZZ, LZ, and HW carried out the human patient tumor tissue analysis. YD made some of the knockout constructs. MZ generated some of the knockout cells and helped with animal experiments. FL helped with carrying out some of the tumor growth experiments and logistics of many experiments. DF and MK generated some of the knockout cells and provided intellectual feedbacks on the manuscript. XL and CL wrote the manuscript.

## Competing Financial Interests

The authors declare no competing financial interests.

## Supplementary Material

Supplementary information, Figure S1 Additional data on DNA damage foci analysis in difference phases of the cell cycle.

Supplementary information, Figure S2 Additional data on cytochrome c leakage from the mitochondria, caspase knockouts, and endoG, CAD knockouts.

Supplementary information, Figure S3 Additional data on the roles of JNK1/2 on cytochrome c leakage and the influence of DNA double strand breaks and activated caspases on the tumorigenic abilities *in vitro* (soft agar) and *in vivo* (xenograft tumor growth).

Supplementary information, Figure S4 More data on the effects of artificially induced DNA DSBs on tumor cell survival *in vitro* or tumor growth *in vivo*.

Supplementary information, Figure S5 The effect of spDSBs ATM activation and the roles of ATM on the tumorigenic abilities of different cancer cells in soft agar assays.

Supplementary information, Figure S6 The influence of caspases, apoptotic endonucleases, and ATM/ATR status on the activation of Stat3 in MDA-MB-231 and MCF7 cells.

Supplementary information, Figure S7 Additional data on the relationship between spDSBs and stemness in patient-derived glioma stem cells.

Supplementary information, Figure S8 Additional data supporting the relevance of the spDSB pathway in human cancer patients.

Supplementary information, Table S1 Primary antibodies used in this study.

Supplementary information, Table S2 Targeting single guided RNA (sgRNA) sequences used in this study

Supplementary information, Table S3 Mutations of target sequences in various CRISPR knockout MDA-MB-231 cancer cells

Supplementary information, Table S4 shRNA sequences of human JNK1 and JNK2
