# Supplementary material for: Self-inflicted DNA double-strand breaks sustain tumorigenicity and stemness of cancer cells
Source: Cell Res. 2017 Mar 24;27(6):764–83. doi: 10.1038/cr.2017.41 (PMC5518870; doi:10.1038/cr.2017.41)
Supplement: Supplementary information, Figure S1 — Additional data on DNA damage foci analysis in difference phases of the cell cycle. [file cr201741x1.pdf]

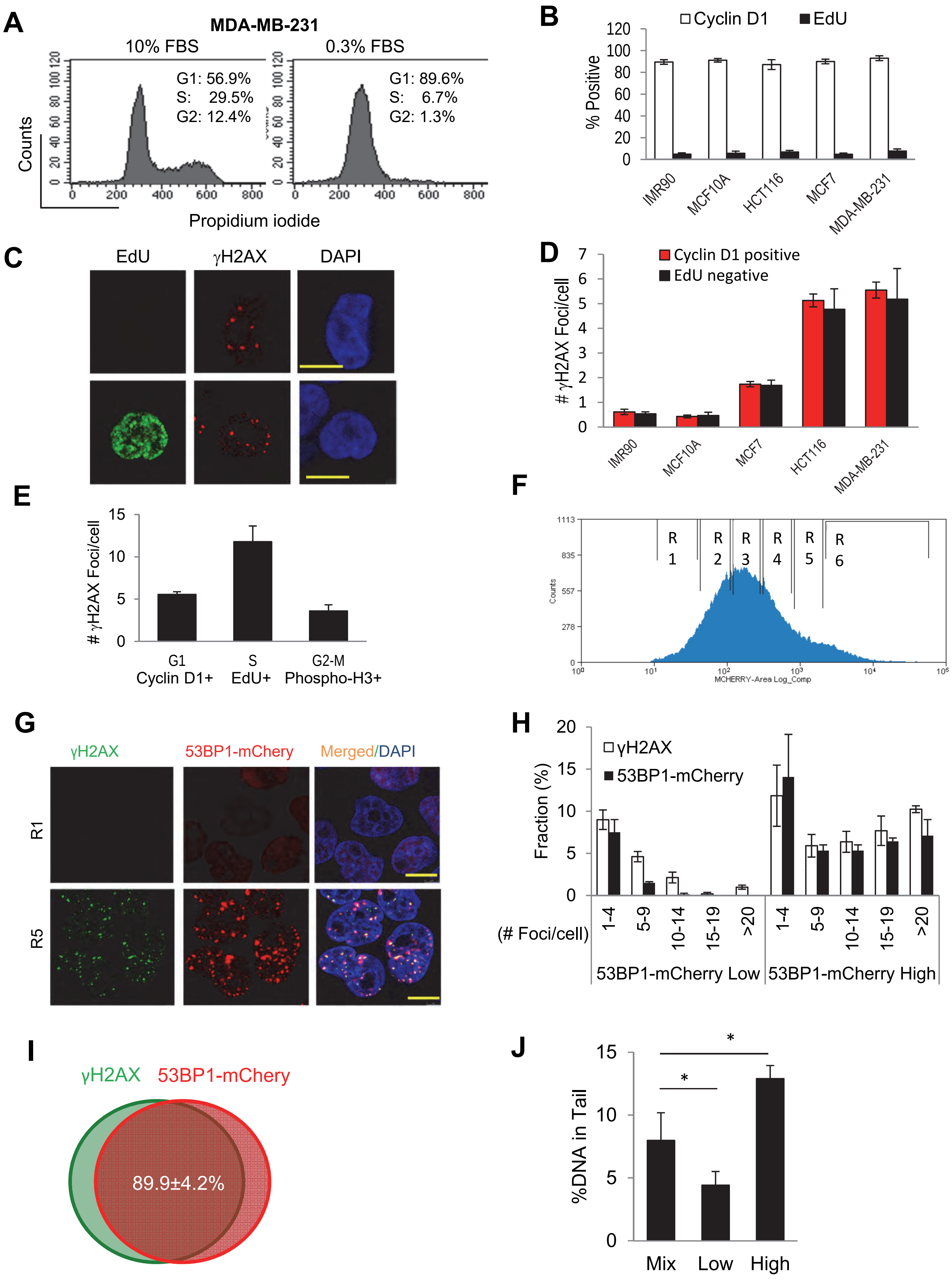
A
MDA-MB-231
10% FBS
0.3% FBS
G1: 56.9%
S: 29.5%
G2: 12.4%
G1: 89.6%
S: 6.7%
G2: 1.3%
Counts
Propidium iodide
B
Cyclin D1
EdU
% Positive
IMR90
MCF10A
HCT116
MCF7
MDA-MB-231
C
EdU
γH2AX
DAPI
D
Cyclin D1 positive
EdU negative
γH2AX Foci/cell
E
γH2AX Foci/cell
G1
Cyclin D1+
S
EdU+
G2-M
Phospho-H3+
F
R 1
R 2
R 3
R 4
R 5
R 6
Counts
MCHERRY-Area Log_Comp
G
γH2AX
53BP1-mChery
Merged/DAPI
R1
R5
H
γH2AX
53BP1-mCherry
Fraction (%)
(# Foci/cell)
1-4
5-9
10-14
15-19
>20
53BP1-mCherry Low
53BP1-mCherry High
I
γH2AX
53BP1-mChery
89.9±4.2%
J
%DNA in Tail
Mix
Low
High

Supplemental information, Figure S1 Additional data on DNA damage foci analysis in difference phases of the cell cycle. (**A**) Flow cytometry analysis of DNA content in MDA-MB-231 cells after serum starvation treatment (0.3% FBS) for 3 days. (**B**) Quantitative analysis the percentage of cells staining positive for cyclin D1 and EdU in various cell lines after serum starvation (0.3% FBS) for 3 days. (**C**) EdU labeling and staining showing existence of γH2AX foci in both EdU+ (S-phase) and EdU-(G1/G2/M) cells. Scale bars indicate 10 μm. (**D**) Quantitative analysis of the average number of γH2AX foci in cyclin D1 positive or EdU negative cells. These data clearly indicate replication stress-independent spDSBs. (**E**) Quantification of the number of γH2AX foci in MDA-MB-231 cells in G1, S, and G2/M phases when cultured exponentially. S phase cells were identified through EdU labeling, G2 were identified though cyclin D1 staining, and G2/M cells were identified through phospho-H3 staining. (**F**) Flow cytometry profiles of 53BP1-mCherry reporter activities in MDA-MB-231 cells . Cells were gated into 6 different groups (R1-R6) and sorted for soft agar and other analysis in subsequent experiments. (**G**) Representative images of γH2AX foci and 53BP1 foci in low(top panels, cells from R1 gate) and high (bottom panels, cells from R5 gate) 53BP1-mCherry expression MDA-MB-231 cells. Scale bar = 25 μm. (**H**) Distribution of γH2AX foci and 53BP1foci in 53BP1-mCherry reporter low and high MDA-MB-231 cells. (**I**) The overlap between γH2AX foci and 53BP1foci in 53BP1-mCherry reporter high MDA-MB-231 cells. (**J**) Comet assay for cells with DNA double strand breaks in 53BP1-mCherry low (R1 in A) and higher expression (R5 in A) MDA-MB-231 cells. *, $p<0.05$. Error bars in B, D, E, H, and J represent standard error of the mean (SEM). n=3.
