# Supplementary material for: Self-inflicted DNA double-strand breaks sustain tumorigenicity and stemness of cancer cells
Source: Cell Res. 2017 Mar 24;27(6):764–83. doi: 10.1038/cr.2017.41 (PMC5518870; doi:10.1038/cr.2017.41)
Supplement: Supplementary information, Figure S2 — Additional data on cytochrome c leakage from the mitochondria, caspase knockouts, and endoG, CAD knockouts. [file cr201741x2.pdf]

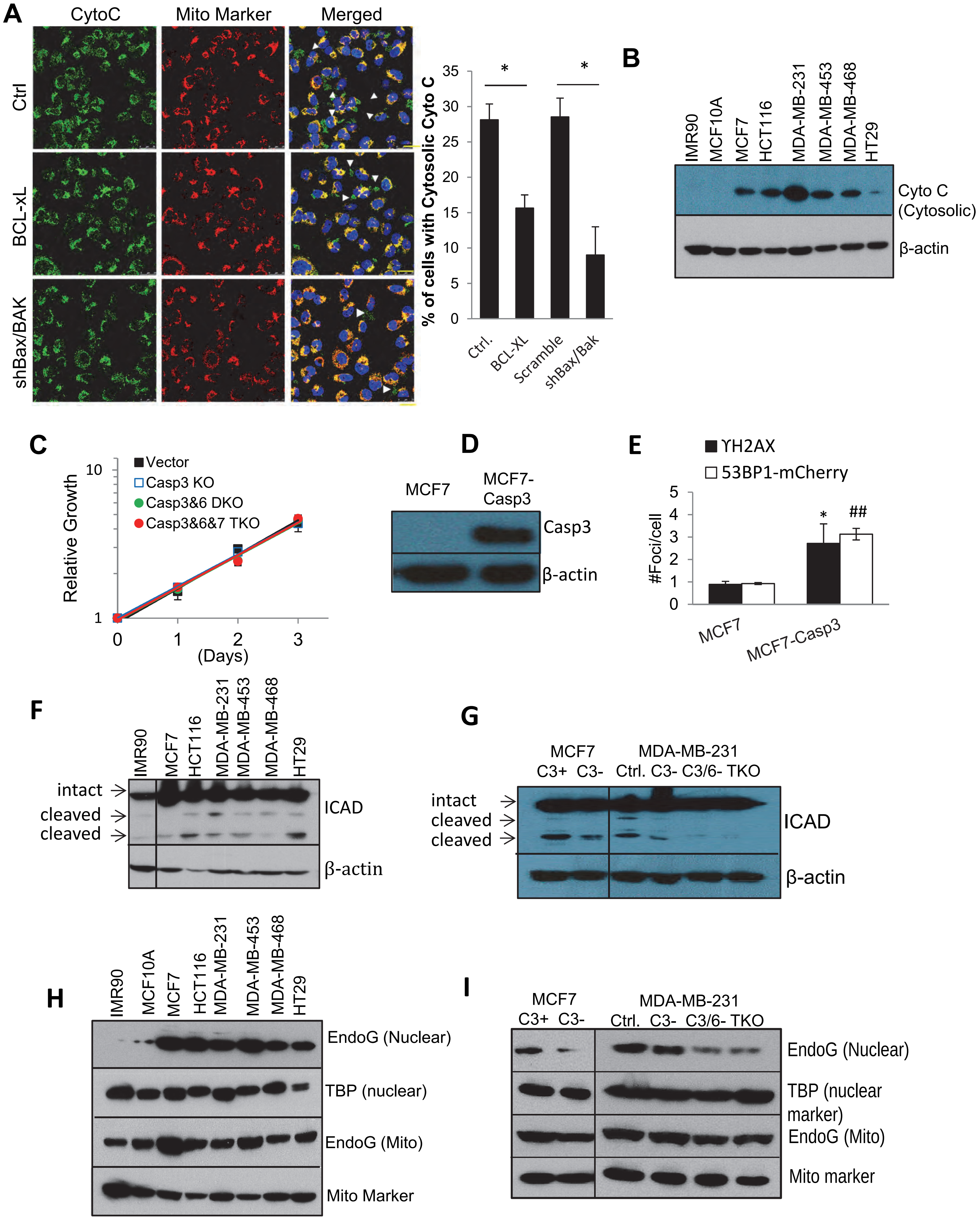
A
CytoC
Mito Marker
Merged
Ctrl
BCL-xL
shBax/BAK
% of cells with Cytosolic Cyto C
35
30
25
20
15
10
5
0
*
*
Ctrl.
BCL-XL
Scramble
shBax/Bak
B
IMR90
MCF10A
MCF7
HCT116
MDA-MB-231
MDA-MB-453
MDA-MB-468
HT29
Cyto C (Cytosolic)
β-actin
C
Vector
Casp3 KO
Casp3&6 DKO
Casp3&6&7 TKO
Relative Growth
10
1
0
1
2
3
(Days)
D
MCF7
MCF7-Casp3
Casp3
β-actin
E
ϒH2AX
53BP1-mCherry
#Foci/cell
5
4
3
2
1
0
*
##
MCF7
MCF7-Casp3
F
IMR90
MCF7
HCT116
MDA-MB-231
MDA-MB-453
MDA-MB-468
HT29
intact
cleaved
cleaved
ICAD
β-actin
G
MCF7
C3+ C3-
MDA-MB-231
Ctrl. C3- C3/6- TKO
intact
cleaved
cleaved
ICAD
β-actin
H
IMR90
MCF10A
MCF7
HCT116
MDA-MB-231
MDA-MB-453
MDA-MB-468
HT29
EndoG (Nuclear)
TBP (nuclear)
EndoG (Mito)
Mito Marker
I
MCF7
C3+ C3-
MDA-MB-231
Ctrl. C3- C3/6- TKO
EndoG (Nuclear)
TBP (nuclear marker)
EndoG (Mito)
Mito marker

Supplemental information, Figure S2 Additional data on cytochrome c leakage from the mitochondria, caspase knockouts, and endoG, CAD knockouts. (**A**) Left panels, confocal imaging of immunofluorescence staining of control and BCL-xL-transduced or shRNA-mediated BAX/BAK double knockdown MDA-MB231 cells. The triangles in the merged panels indicate some of the cells with extra-mitochondrial cytochrome C. Scale bar = 25 μm. Right panel, quantitative estimate of fraction of cells with extra-mitochondrial cytochrome C. Significant difference exists between the two groups, $p<0.05$. Error bars represent SEM, n=3. (**B**) Western blot analysis cytochrome c levels in the extra-mitochondrial, cytosolic fractions of various cells. (**C**) Growth curve of MDA-MB-231 cells with various caspase knockouts. (**D**) Western blot analysis of Casp3 expression in wild type MCF7 cells and MCF7-Casp3, which has an exogenously expressed *CASP3* gene with an HA tag. (**E**) Quantitative data for γH2AX and 53BP1-mCherry foci in parental MCF7 cells and those with exogenous Casp3 expression (MCF7-Casp3). Parental MCF7 cells are deficient in Caspase-3 expression. The error bars shows SEM, n=3, *,p=0.0219; ##,p=0.0015, Student's t-test. (**F**) Western blot analysis full length (top band) and cleavage products (lower two bands) ICAD in normal and tumor cell lines. (**G**) Western blot analysis of ICAD cleavage in control and caspase knockout MDA-MB-231 cells lines and MCF7 cells with or without exogenous Casp3 gene expression. (**H**) Western blot analysis EndoG in nuclear and mitochondria fractions of various cell lines. TATA binding protein(TBP), and mito marker were used as nuclear and mitochondria loading controls, respectively. (**I**) Western blot analysis of EndoG in nuclear and mitochondrial fractions of control and caspase knockout MDA-MB-231 cells lines and MCF7 cells with or without exogenous Casp3 gene expression.
