# Supplementary material for: Self-inflicted DNA double-strand breaks sustain tumorigenicity and stemness of cancer cells
Source: Cell Res. 2017 Mar 24;27(6):764–83. doi: 10.1038/cr.2017.41 (PMC5518870; doi:10.1038/cr.2017.41)
Supplement: Supplementary information, Figure S3 — Additional data on the roles of JNK1/2 on cytochrome c leakage and the influence of DNA double strand breaks and activated caspases on the tumorigenic abilities in vitro (soft agar) and in vivo (xenograft tumor growth). [file cr201741x3.pdf]

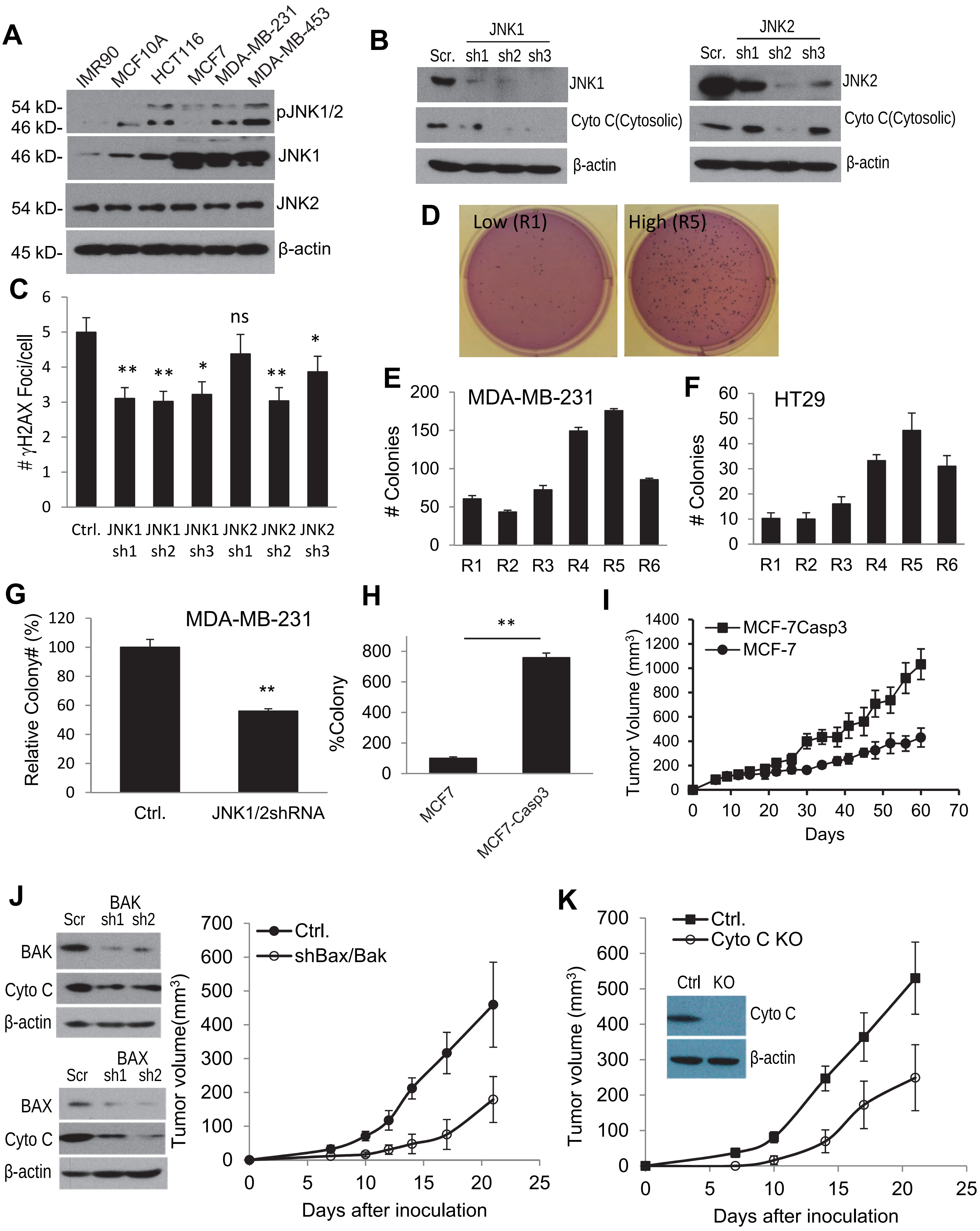

A
IMR90
MCF10A
HCT116
MCF7
MDA-MB-231
MDA-MB-453
54 kD-
46 kD-
pJNK1/2
46 kD-
JNK1
54 kD-
JNK2
45 kD-
β-actin
B
JNK1
Scr.
sh1
sh2
sh3
JNK1
Cyto C(Cytosolic)
β-actin
JNK2
Scr.
sh1
sh2
sh3
JNK2
Cyto C(Cytosolic)
β-actin
C
γH2AX Foci/cell
ns
Ctrl.
JNK1 sh1
JNK1 sh2
JNK1 sh3
JNK2 sh1
JNK2 sh2
JNK2 sh3
D
Low (R1)
High (R5)
E
MDA-MB-231
Colonies
R1
R2
R3
R4
R5
R6
F
HT29
Colonies
R1
R2
R3
R4
R5
R6
G
MDA-MB-231
Relative Colony# (%)
Ctrl.
JNK1/2shRNA
H
%Colony
MCF7
MCF7-Casp3
I
MCF-7Casp3
MCF-7
Tumor Volume (mm3)
Days
J
BAK
Scr
sh1
sh2
BAK
Cyto C
β-actin
BAX
Scr
sh1
sh2
BAX
Cyto C
β-actin
Ctrl.
shBax/Bak
Tumor volume(mm3)
Days after inoculation
K
Ctrl.
Cyto C KO
Ctrl
KO
Cyto C
β-actin
Tumor volume (mm3)
Days after inoculation

Supplemental information, Figure S3 Additional data on the roles of JNK1/2 on cytochrome c leakage and the influence of DNA double strand breaks and activated caspases on the tumorigenic abilities *in vitro* (soft agar) and *in vivo* (xenograft tumor growth). (**A**) Constitutive activation of JNK1 and JNK2 in various cancer cells. An antibody that can detect the phosphorylated forms both of JNK1 and JNK2 were used the detect JNK1/2 activation. (**B**) ShRNA mediated depletion of JNK1 and JNK2 demonstrating the important roles of JNK1 and JNK2 in extra-mitochondrial leakage of cytochrome c in MDA-MB-231 cells. (**C**) Significant reduction in γH2AX foci formation in MDA-MB-231 cells with depleted JNK1 and JNK2. Student's t test was used. Ctrl. vs other group, *, $p<0.01$, **, $p<0.001$, ns, $p=0.094$. (**D**) Representative images of soft agar colony growth from MDA-MB-231 cells with low and higher 53BP1-mCherry reporter expression. (**E**) Average number of soft agar colony formation from MDA-MB-231 cells sorted according to their 53BP1-mCherry reporter expression status (see Fig. S1A). (**F**) Soft agar colony forming abilities of HT29 cells from different 53BP1-mCherry expression levels (following the same procedure as shown in Fig. S1A) as sorted by use of an FACS sorter. (**G**) Significant reduction in soft agar colony formation in MDA-MB-231 cells with JNK1/ JNK2 depletion (JNK1-sh2 + JNK2-sh2). **, $p<0.001$, n=3, Student's t test. (**H**) Soft agar colony growth from parental MCF7 and MCF7-Casp3 cells. About 5000 cells were seeded in each well in 6-well plates. **, $p<0.001$, Students t-test, n=3. Error bars represent SD. (**I**) Tumor growth rate were significantly increased in MCF7 cells with exogenous Casp3 expression (MCF7-Casp3). MCF7 cells are Casp3-deficient. Error bars represent SEM, $p=0.02$ at the end of the experiments, n=10, Student *t* test. (**J**) ShRNA-mediated knockdown of BAK and BAX in MDA-MB231 cells and their influence on the tumorigenicity. Western blot analysis (left panel) showed that shRNA1 for BAK and shRNA2 for BAX appeared to be more efficacious in knocking down their targets and reduce cytosolic cyto C leakage. These two shRNAs were then used to carry out a double knockdown of BAX/BAK in MDA-MD231 cells. About 5×10^5 MDA-MB231 cells were injected subcutaneously in SCID mice. Tumor sizes were then measured periodically. The difference between the two groups are significant, *$p<0.05$ when comparing the last day of measurements, n=6, Student' t-test, error bars represent SEM. (**K**) Tumor growth from MDA-MB231 cell with cytochorme C knockout (see inset for western blot analysis). About 5×10^5 MDA-MB231 cells were injected subcutaneously in SCID mice. Tumor sizes were then measured periodically. The difference between the two groups are statistically significant, *$p<0.05$ when comparing the last day of measurement, n=5, Student' t-test, error bars represent SEM. Error bars in C, E, F, and G represent standard error of the mean, n=3.
