# Supplementary material for: Self-inflicted DNA double-strand breaks sustain tumorigenicity and stemness of cancer cells
Source: Cell Res. 2017 Mar 24;27(6):764–83. doi: 10.1038/cr.2017.41 (PMC5518870; doi:10.1038/cr.2017.41)
Supplement: Supplementary information, Figure S4 — More data on the effects of artificially induced DNA DSBs on tumor cell survival in vitro or tumor growth in vivo. [file cr201741x4.pdf]

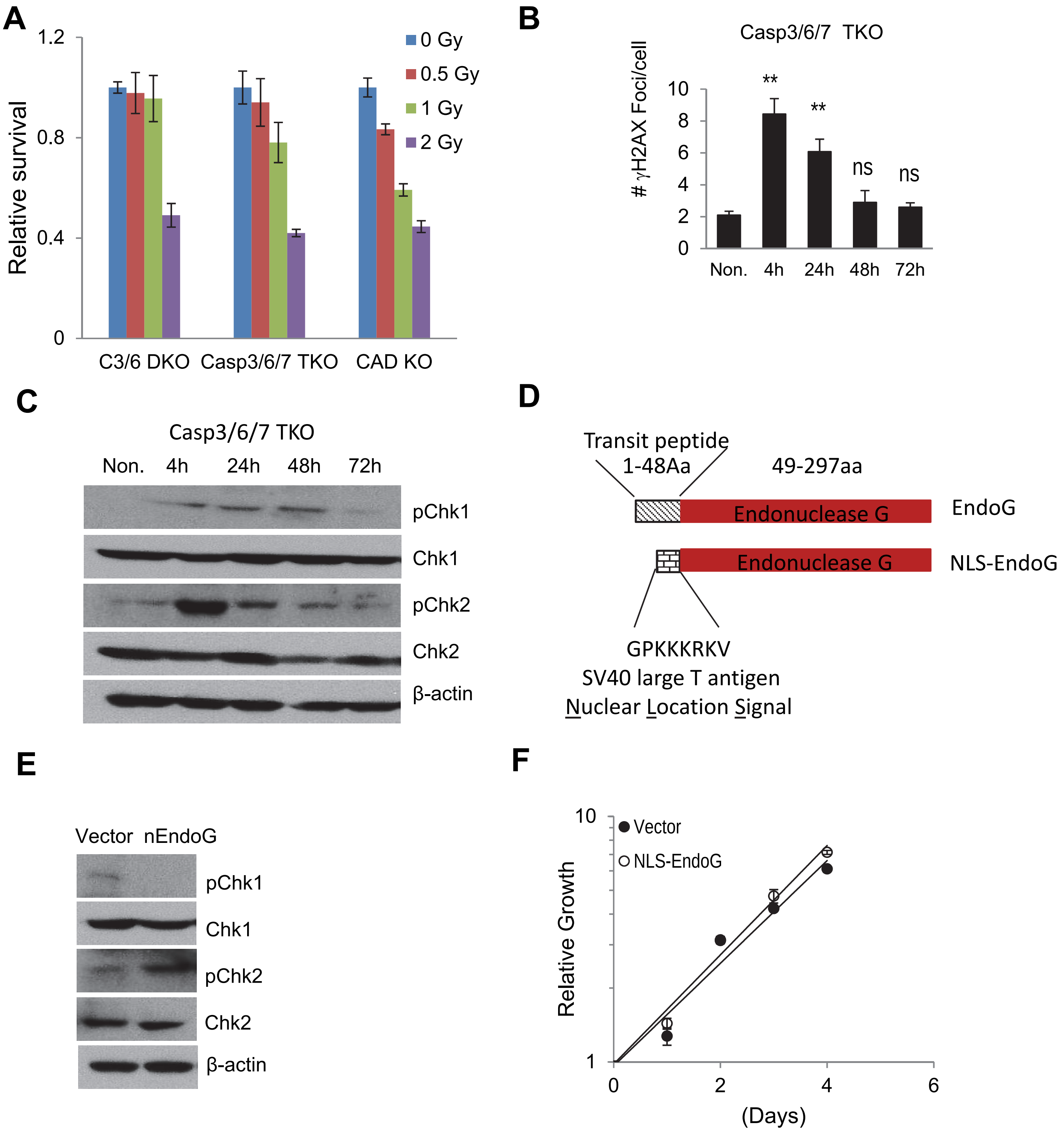
A
B
C
D
E
F
1.2
0.8
0.4
0
Relative survival
0 Gy
0.5 Gy
1 Gy
2 Gy
C3/6 DKO
Casp3/6/7 TKO
CAD KO
Casp3/6/7 TKO
γH2AX Foci/cell
10
8
6
4
2
0
**
**
ns
ns
Non.
4h
24h
48h
72h
Casp3/6/7 TKO
Non.
4h
24h
48h
72h
pChk1
Chk1
pChk2
Chk2
β-actin
Transit peptide
1-48Aa
49-297aa
Endonuclease G
EndoG
Endonuclease G
NLS-EndoG
GPKKKRKV
SV40 large T antigen
Nuclear Location Signal
Vector nEndoG
pChk1
Chk1
pChk2
Chk2
β-actin
10
1
Vector
NLS-EndoG
Relative Growth
0
2
4
6
(Days)

Supplemental information, Figure S4 More data on the effects of artificially induced DNA DSBs on tumor cell survival *in vitro* or tumor growth *in vivo*. (**A**) Relative survival of low dose X-Ray treated CRISPR/CAS9-mediated knockout MDA-MB-231 cells. (**B**) Quantitative estimate of γH2AX foci formation in Casp3/6/7 TKO cells exposed to 0.8 Gy of x-rays. Error bars represent standard error of the mean (SEM). Student's t test was used. **, $p<0.001$, irradiated vs non-irradiated groups; ns, $p>0.05$. (**C**) Western blot analysis of phosphorylated Chk1 and Chk2 in Casp3/6/7-TKO cells exposed to 0.8 Gy of x-rays. (**D**) Diagram of modified *ENDOG* . The transit peptide sequence of original *ENDOG* gene was replaced by nuclear location signal sequence (*NLS-ENDOG*). (**E**) Western blot analysis of phosphorylated Chk1 and Chk2 in vector and NLS-EndoG transduced MDA-MB-231 cells. (**F**) Growth curve for vector and NLS-EndoG transduced MDA-MB-231 cells.
