# Supplementary material for: Self-inflicted DNA double-strand breaks sustain tumorigenicity and stemness of cancer cells
Source: Cell Res. 2017 Mar 24;27(6):764–83. doi: 10.1038/cr.2017.41 (PMC5518870; doi:10.1038/cr.2017.41)
Supplement: Supplementary information, Figure S5 — The effect of spDSBs ATM activation and the roles of ATM on the tumorigenic abilities of different cancer cells in soft agar assays. [file cr201741x5.pdf]

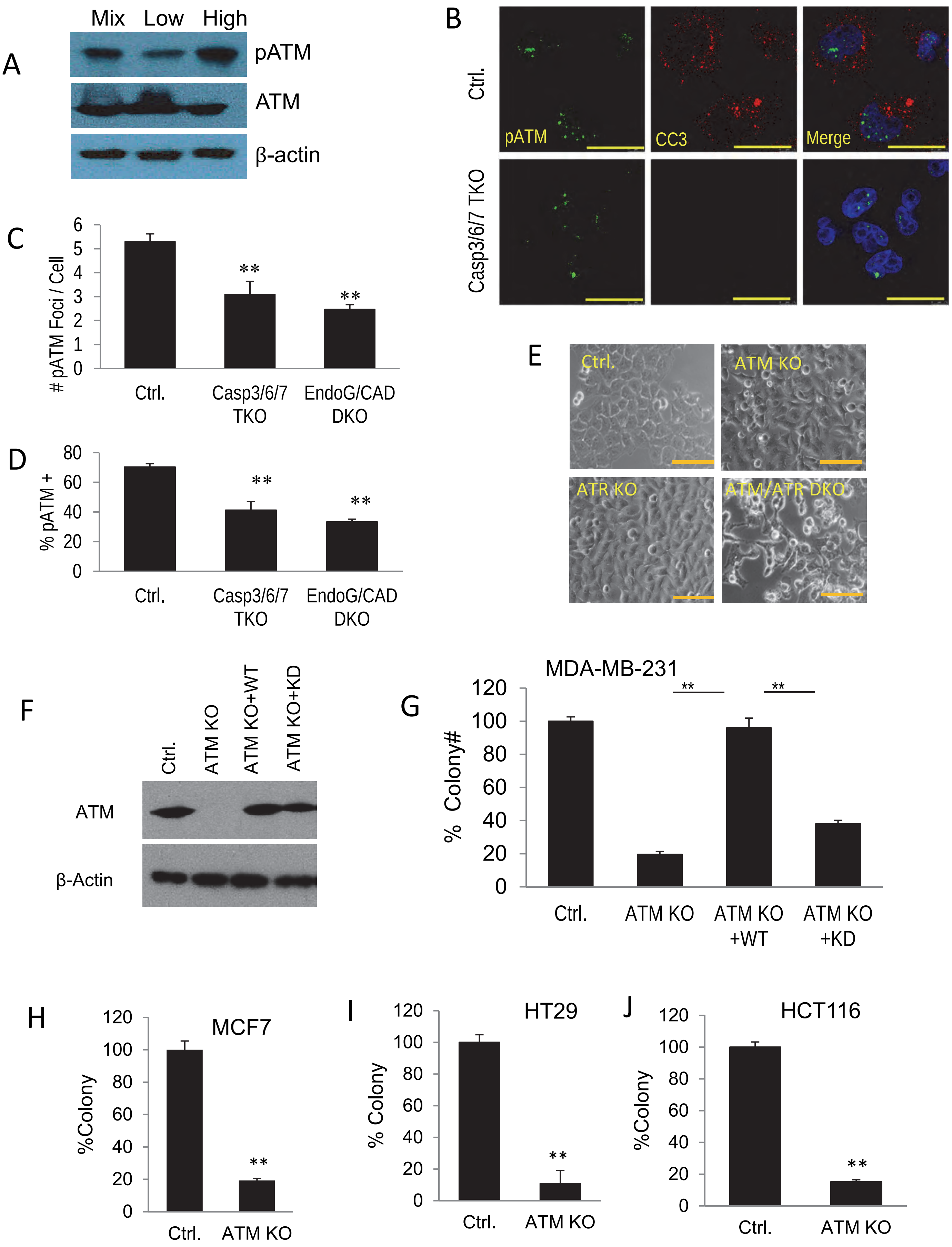

Soft agar colony growth

Supplemental information, Figure S5 The effect of spDSBs ATM activation and the roles of ATM on the tumorigenic abilities of different cancer cells in soft agar assays. (**A**) Western blot analysis of phosphorylated and total ATM expression in low and high 53BP1-mCherry expressing MDA-MB-231 cells. (**B**) Confocal microscope imaging of cleaved caspase3 (CC3) and phosphorylated ATM in MDA-MB-231 cells with or without Casp3/6/7 triple knockout. Scale bar= 20 μm. (**C**) The average number of phosphorylated ATM (pATM) foci in MDA-MB-231 cells with or without Casp3/6/7 TKO and endoG/CAD DKO. Ctrl. vs other group, **, $p<0.001$. (**D**) Fraction of pATM positive cells in MDA-MB-231 cells with or without Casp3/6/7 TKO and endoG/CAD DKO. Ctrl. vs other groups, **, $p<0.001$. (**E**) Typical morphology of CRISPR/Cas9-mediated ATM KO, ATR KO and ATM/ATR DKO MDA-MB-231 cells. (Scale bar=100μm). (**F**) Western blot confirmation of restored wild type (WT) and catalytically inactive (kinase dead) versions of ATM (D2870A N2875K) in MDA-MB231 cells in MDA-MB231 ATMKO cells. (**G**) Soft agar colony formation in MDA-MB-231 cells with restored ATM expression. It is clear that wild type ATM expression restored almost all of the soft agar forming abilities of MDA-MB-231 ATMKO cells. On the other hand ATM-KD expression was not able to fully restore the tumorigencity of MDA-MB231KO cells. These results demonstrate the importance of the kinase activities of ATM for the tumorigenicity of MDA-MB-231 cells. (**H**) Soft agar colony formation abilities of control and *ATM* KO MCF7 breast cancer cells. (**I**) Soft agar colony formation abilities of control and *ATM* KO HT29 colon cancer cells. (**J**) Soft agar colony formation abilities of control and *ATM* KO HCT116 colon cancer cells. Error bars in C, D, G-J represent SEM, n=3 ; **, $p<0.001$, Student's t-test.
