# Supplementary material for: Self-inflicted DNA double-strand breaks sustain tumorigenicity and stemness of cancer cells
Source: Cell Res. 2017 Mar 24;27(6):764–83. doi: 10.1038/cr.2017.41 (PMC5518870; doi:10.1038/cr.2017.41)
Supplement: Supplementary information, Figure S6 — The influence of caspases, apoptotic endonucleases, and ATM/ATR status on the activation of Stat3 in MDA-MB-231 and MCF7 cells. [file cr201741x6.pdf]

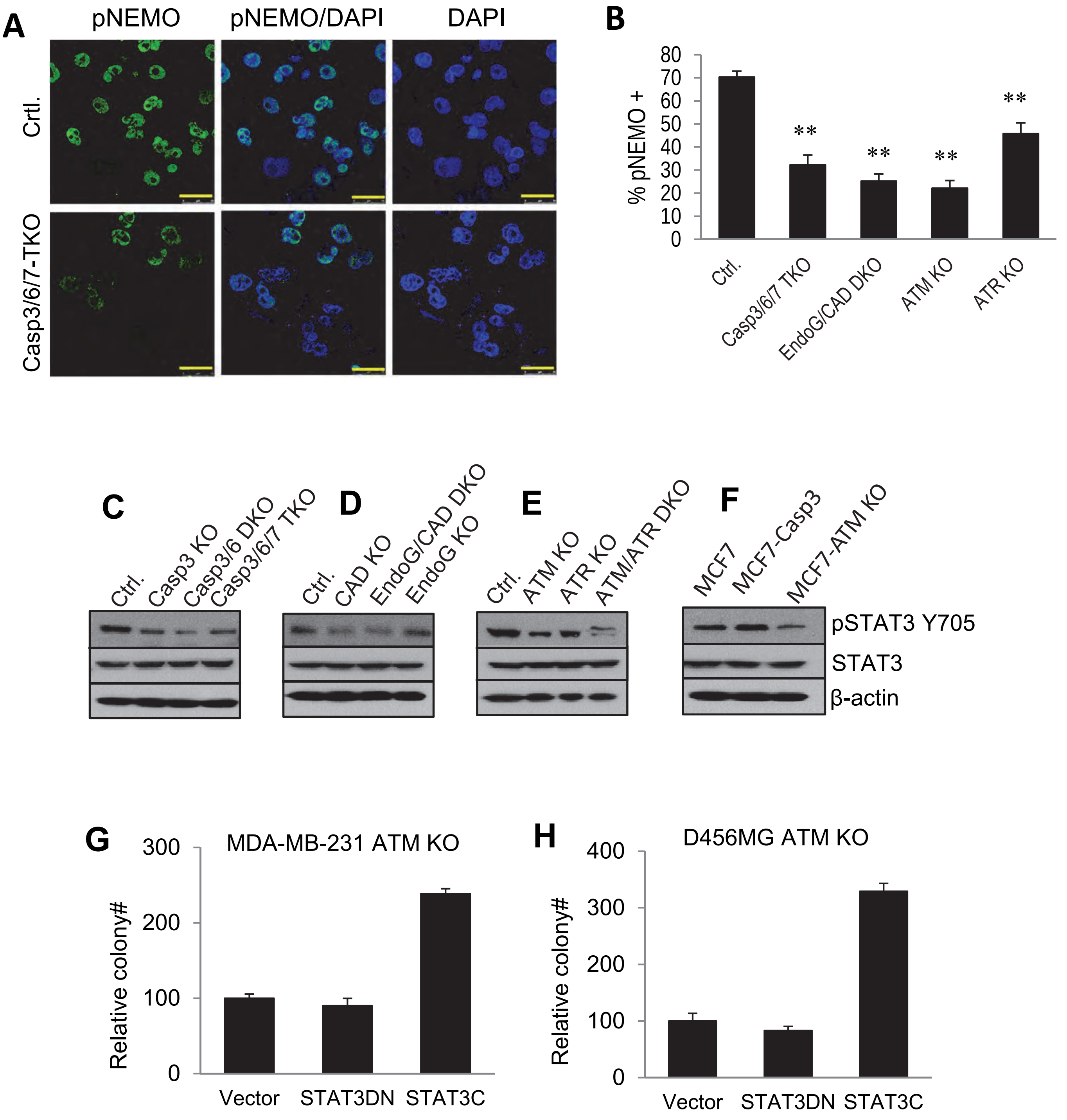
A
pNEMO
pNEMO/DAPI
DAPI
Crtl.
Casp3/6/7-TKO
B
% pNEMO +
80
70
60
50
40
30
20
10
0
**
**
**
**
Ctrl.
Casp3/6/7 TKO
EndoG/CAD DKO
ATM KO
ATR KO
C
Ctrl.
Casp3 KO
Casp3/6 DKO
Casp3/6/7 TKO
D
Ctrl.
CAD KO
EndoG/CAD DKO
EndoG KO
E
Ctrl.
ATM KO
ATR KO
ATM/ATR DKO
F
MCF7
MCF7-Casp3
MCF7-ATM KO
pSTAT3 Y705
STAT3
β-actin
G
MDA-MB-231 ATM KO
Relative colony#
300
200
100
0
Vector
STAT3DN
STAT3C
H
D456MG ATM KO
Relative colony#
400
300
200
100
0
Vector
STAT3DN
STAT3C

**Supplemental information, Figure S6** The influence of caspases, apoptotic endonucleases, and ATM/ATR status on the activation of Stat3 in MDA-MB-231 and MCF7 cells. (**A**) Confocal microscope imaging of phosphorylated NEMO Ser85(pNEMO) in MDA-MB-231cells with or without Casp3/6/7TKO. Scale bar= 20 μm. (**B**) Fraction of cells with phosphorylated NEMO in MDA-MB-231 cells with or without Casp3/6/7 TKO, EndoG/CAD DKO, ATM KO, or ATR KO . Error bars represent standard error of the mean (SEM). Student's t test was used. Ctrl. vs other group, **, $p<0.001$. (**C**) Western blot analysis of phosphorylated STAT3 expression in vector-transduced control, and caspase-3,-6,-7 knockout MDA-MB-231 cells. (**D**) Western blot analysis of phosphorylated STAT3 expression in vector-transduced control, EndoG, and CAD knockout MDA-MB-231 cells. (**E**) Western blot analysis of phosphorylated STAT3 expression in vector-transduced control, ATM, and ATR knockout MDA-MB-231 cells. (**F**) Western blot analysis of phosphorylated STAT3 expression in parental MCF7, MCF7-*CASP3*, and MCF7-*ATMKO* cells. (**G**) Soft agar colony forming assay from MDA-MB-231 ATMKO cells that were exogenously transduced with vector control, dominant negative STAT3DN (Y705F), and constitutively active STAT3C (A661C, N663C). Only STAT3C was able to enhance the low soft agar colony forming abilities of MDA-MB231 ATMKO cells. (**H**) Soft agar colony forming assay from D456MG ATMKO glioma cells that were exogenously transduced with vector control, dominant negative STAT3DN (Y705F), and constitutively active STAT3C (A661C, N663C). Only STAT3C was able to enhance the low soft agar colony forming abilities of D456MG ATMKO cells
