# Supplementary material for: Self-inflicted DNA double-strand breaks sustain tumorigenicity and stemness of cancer cells
Source: Cell Res. 2017 Mar 24;27(6):764–83. doi: 10.1038/cr.2017.41 (PMC5518870; doi:10.1038/cr.2017.41)
Supplement: Supplementary information, Figure S7 — Additional data on the relationship between spDSBs and stemness in patient-derived glioma stem cells. [file cr201741x7.pdf]

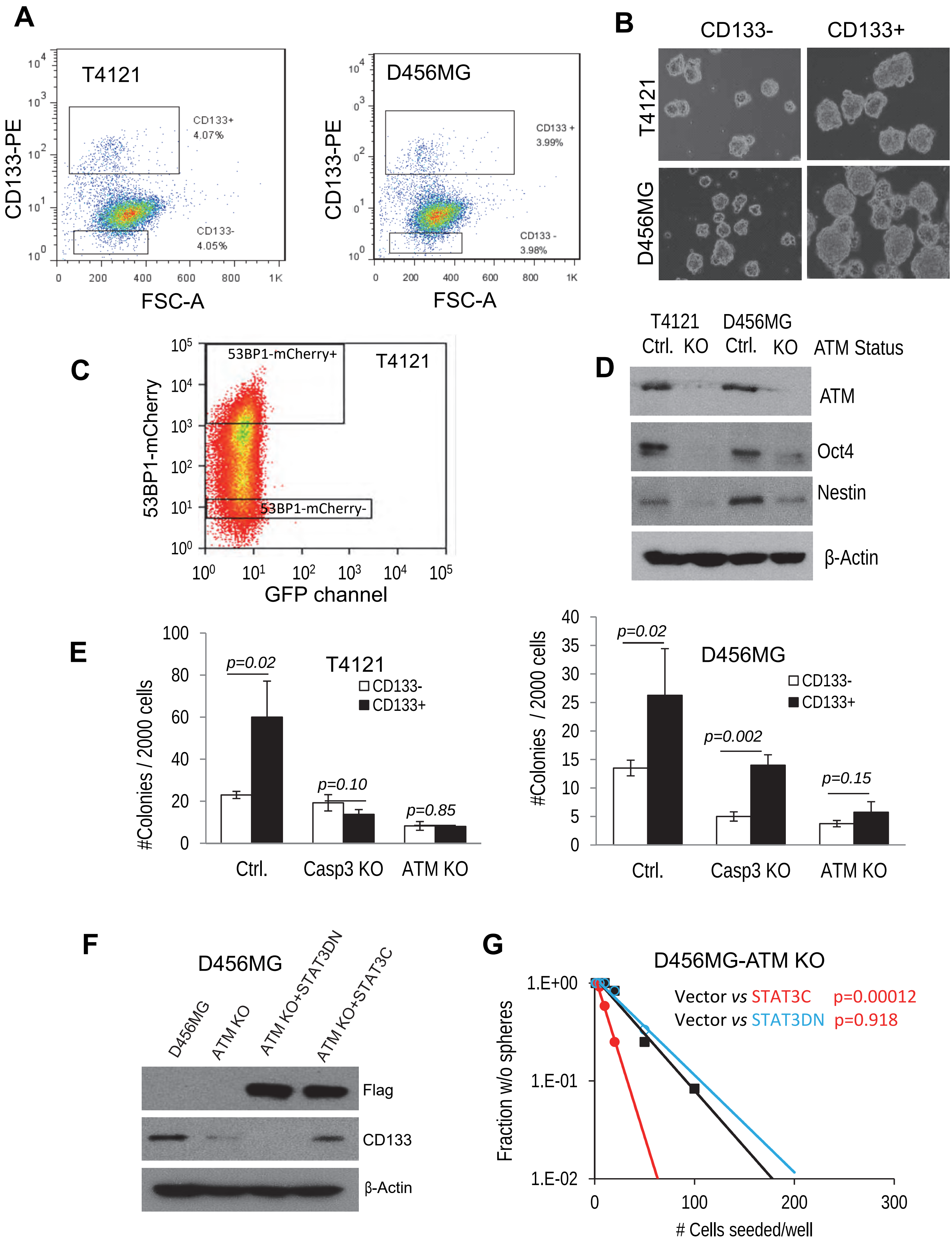

A
T4121
CD133-PE
CD133+
4.07%
CD133-
4.05%
FSC-A
D456MG
CD133 +
3.99%
CD133 -
3.98%
B
CD133-
CD133+
T4121
D456MG
C
53BP1-mCherry+
T4121
53BP1-mCherry
53BP1-mCherry-
GFP channel
D
T4121
D456MG
Ctrl. KO Ctrl. KO
ATM Status
ATM
Oct4
Nestin
β-Actin
E
T4121
#Colonies / 2000 cells
p=0.02
p=0.10
p=0.85
CD133-
CD133+
Ctrl.
Casp3 KO
ATM KO
D456MG
p=0.02
p=0.002
p=0.15
F
D456MG
ATM KO
ATM KO+STAT3DN
ATM KO+STAT3C
Flag
CD133
β-Actin
G
D456MG-ATM KO
Vector vs STAT3C p=0.00012
Vector vs STAT3DN p=0.918
Fraction w/o spheres
Cells seeded/well

Supplemental information, Figure S7 Additional data on the relationship between spDSBs and stemness in patient-derived glioma stem cells. (**A**) Selection criteria for the sorting of CD133 positive T4121 , and D456MG patient-derived glioma cells. (**B**) Tumor sphere formation from sorted CD133 negative (CD133-) and CD133 positive (CD133+) T4121 & D456MG glioma cells. (**C**) Selection criteria for the sorting of 53BP1-mCherry-high(+) and 53BP1-mCherry-low(-) T4121 patient-derived glioma cells. (**D**) Western blot analysis stem cell markers OCT4, Nestin in ATM KO glioma cells. (**E**) Soft agar colony forming abilities of T4121 and D456MG glioma cells with ATM and CASP3 gene knockouts. CD133 positive and negative cells were sorted from each knockout line and evaluated separately for their ability to form soft agar colonies. (**F**) Western blot analysis glioma stem cell marker CD133 expression in D456MG ATMKO cells with exogenous expression of constitutively active STAT3C (A661C, N663C) and dominant-negative STAT3DN (Y705F) expression. Exogenous expression of STAT3s were detected by use of a Flag tag. Only STAT3C were able to restore CD133 expression that is missing in D456MG ATMKO cells. (**G**) Limited dilution analysis of tumor sphere formation in D456MG-ATMKO cells. Black line: D456MG-ATMKO cells transduced with vector control. Red line, D456MG-ATMKO cells transduced with STAT3C. Blue line, D456MG cells transduced with STAT3DN. It is clear that STAT3C was able to enhance to reduced tumor sphere forming abilities of D455MG-ATMKO cells but not STAT3DN. P values were calculated from chi-square test.
