# Supplementary material for: Self-inflicted DNA double-strand breaks sustain tumorigenicity and stemness of cancer cells
Source: Cell Res. 2017 Mar 24;27(6):764–83. doi: 10.1038/cr.2017.41 (PMC5518870; doi:10.1038/cr.2017.41)
Supplement: Supplementary information, Figure S8 — Additional data supporting the relevance of the spDSB pathway in human cancer patients. [file cr201741x8.pdf]

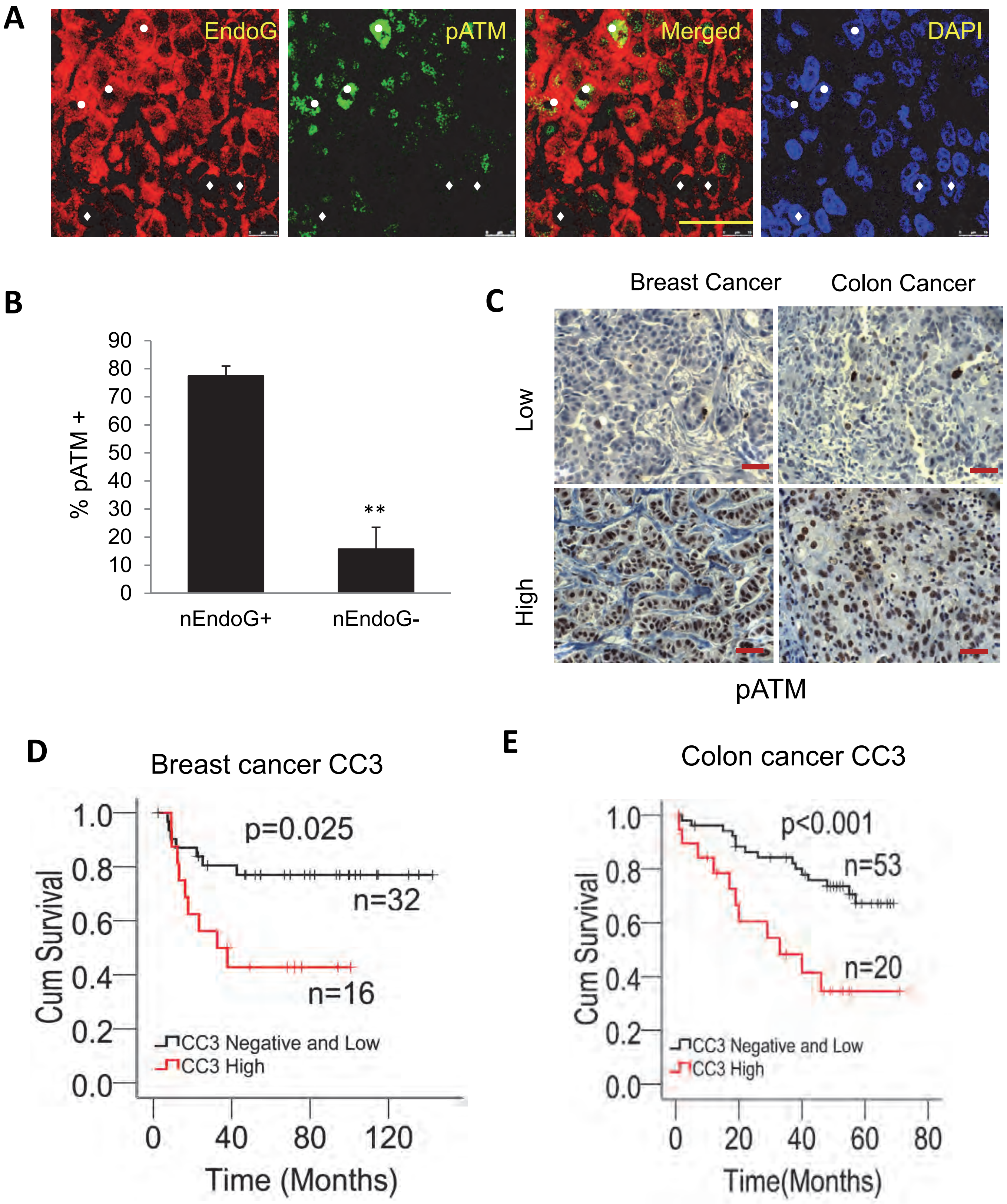
A
EndoG
pATM
Merged
DAPI
B
% pATM +
90
80
70
60
50
40
30
20
10
0
**
nEndoG+
nEndoG-
C
Breast Cancer
Colon Cancer
Low
High
pATM
D
Breast cancer CC3
p=0.025
n=32
n=16
Cum Survival
CC3 Negative and Low
CC3 High
Time (Months)
E
Colon cancer CC3
p<0.001
n=53
n=20
Cum Survival
CC3 Negative and Low
CC3 High
Time(Months)

Supplemental information, Figure S8, Additional data supporting the relevance of the spDSB pathway in human cancer patients. (**A**) Confocal microscope images of immunofluorescence co-staining of endonucleaes G with pATM in human breast cancer tissue. Circular dots in the images highlight examples of cells with positive nuclear endoG staining and pATM staning while diamond dots highlight examples of cells with no nuclear endoG staining. Scale bar: 25μm. (**B**) Quantitative estimate of the fraction of cells that stained positive for pATM among cells with (nEndoG+) or without (nEndoG-) nuclear endoG. **, $p<0.001$, $n=3$, Student's t-test. Error bars represent standard error of the mean (SEM). (**C**) Representative IHC images of pATM staining showing low (top panel) and high (lower panel) levels of pATM expression in breast cancer patients. Scale bar=100μm. (**D**) Kaplan-Meier survival analysis of a cohort of breast cancer patients. Plotted is patient survival for those with high or low levels of cleaved caspase3 in their pre-treatment tumor samples. (**E**) Kaplan-Meier survival analysis of a cohort of colon cancer patients. Plotted is patient survival for those with high or low levels of cleaved caspase3 in their pre-treatment tumor samples.
