# Supplementary material for: Self-inflicted DNA double-strand breaks sustain tumorigenicity and stemness of cancer cells
Source: Cell Res. 2017 Mar 24;27(6):764–83. doi: 10.1038/cr.2017.41 (PMC5518870; doi:10.1038/cr.2017.41)
Supplement: Supplementary information, Table S1 — Primary antibodies used in this study. [file cr201741x9.pdf]

Table S1. Primary antibodies used in this study.

Target protein	Antibody source	Clone information
γH2AX (Ser139)	Upstate Biotechnology	JBW301, Mouse mAb
Caspase-3 (full length)	Cell Signaling Technology	8G10 , Rabbit mAb
Caspase-3 (cleaved,Asp175)	Cell Signaling Technology	5A1E, Rabbit mAb
Caspase-6 (full length)	Cell Signaling Technology	Rabbit polyclonal
Caspase-6 (cleaved, Asp162)	Cell Signaling Technology	Rabbit polyclonal
Caspase-7 (full length)	Cell Signaling Technology	D2Q3L, Rabbit mAb
Caspase-7 (cleaved, Asp198)	Cell Signaling Technology	D6H1, Rabbit mAb
Cytochrome C	Abcam	EPR1327, Rabbit mAb
EndoG	Chemicon	Rabbit polyclonal
EndoG	Origene	OTI5F1, Mouse monoclonal
CAD	Santa Cruz biotechnology	FL-338, Rabbit polyclonal
ICAD	Santa Cruz biotechnology	FL-331, Rabbit polyclonal
HA epitope	Novus Biologicals	Goat polyclonal
ATM	Cell Signaling Technology	D2E2, Rabbit mAb
Phospho-ATM (S1981)	Abcam	EP1890Y, Rabbit mAb
ATR	Cell Signaling Technology	Rabbit polyclonal
Phospho-ATR(Ser428)	Cell Signaling Technology	Rabbit polyclonal
IKKϒ (Nemo)	BD Pharrmingen	Mouse monoclonal
Phospho-IKKϒ (Ser85)	LifeSpan Biosciences	Rabbit polyclonal
NFκB p65	Cell Signaling Technology	D14E12, Rabbit mAb
Phospho-NFκB p65(Ser536)	Cell Signaling Technology	93H1, Rabbit mAb
STAT3	Cell Signaling Technology	Rabbit polyclonal
Phospho-STAT3 Y705	Cell Signaling Technology	Rabbit polyclonal
JNK1	Abcam	Rabbit polyclonal
JNK2	Abcam	EP1595Y, Rabbit mAb
Bax	Cell Signaling Technology	D2E11, Rabbit mAb
Bak	Cell Signaling Technology	D4E4, Rabbit mAb
OCT3/4	Santa Cruz Biotechnology	N-19, Goat polyclonal
Nestin	Millipore	Mouse monoclonal
Phospho-JNK1/2	Cell Signaling Technology	81E11, Rabbit mAb
Phospho-Chk1 (Ser345)	Cell Signaling Technology	133D3, Rabbit mAb
Phospho-Chk2 (Thr68)	Novus	Rabbit polyclonal
β-Actin	Novus Biologicals	Mouse mAb
