# Supplementary material for: Self-inflicted DNA double-strand breaks sustain tumorigenicity and stemness of cancer cells
Source: Cell Res. 2017 Mar 24;27(6):764–83. doi: 10.1038/cr.2017.41 (PMC5518870; doi:10.1038/cr.2017.41)
Supplement: Supplementary information, Table S2 — Targeting single guided RNA (sgRNA) sequences used in this study [file cr201741x10.pdf]

Table S2. Targeting single guided RNA (sgRNA) sequences used in this study

Gene	Accession	sgRNA oligo(5`-3`)*	Targeted Exon
CASPASE3	NC_000004	CACCGcatacatggaagcgaatcaa<br>AAACttgattcgcttccatgtatgC	Exon4
CASPASE3	NC_000004	CACCGggaagcgaatcaatggactc<br>AAACgagtccattgattcgcttccC	Exon4
CASPASE6	NC_000004	CACCaagattgtctctatctgcgcGT<br>TAAAACgcgcagatagagacaatctt	Exon2
CASPASE6	NC_000004	CACCgcgcaggtgccccgcctttcGT<br>TAAAACgaaaggcggggcacctgcgc	Exon2
CAD	NC_000001	CACCGcagcccgaggaagttcggcg<br>AAACcgccgaacttcctcgggctgC	Exon1
CAD	NC_000001	CACCGgcccgcagcttcacgctctt<br>AAACaagagcgtgaagctgcgggcC	Exon1
EndoG	NC_000009	CACCGgggctgggtgcggtcgtcga<br>AAACtcgacgaccgcacccagcccC	Exon1
EndoG	NC_000009	CACCGcgacttccgcgaggacgact<br>AAACagtcgtcctcgcggaagtcgC	Exon1
CASPASE7	NC_000010	CACCggtacaaacgaggaccggtcGT<br>TAAAACgaccggtcctcgtttgtacc	Exon2
CASPASE7	NC_000010	CACCgggacggtacaaacgaggacGT<br>TAAAACgtcctcgtttgtaccgtccc	Exon2
ATM	NC_000011	CACCGttgtttcaggatctcgaatc<br>AAACgattcgagatcctgaaacaaC	Exon3
ATM	NC_000011	CACCGcggcattcagattccaaaca<br>AAACtgtttggaatctgaatgccgC	Exon3
ATR	NC_000003	CACCGgaaatcaagcaacatcacgg<br>AAACccgtgatgttgcttgatttcC	Exon3
ATR	NC_000003	CACCGgggaggatttcatgatatgc<br>AAACgcatatcatgaaatcctcccC	Exon3

*Capital letters: enzyme overhangs; non-capital letters: sgRNA target guide sequence
