# Supplementary material for: Self-inflicted DNA double-strand breaks sustain tumorigenicity and stemness of cancer cells
Source: Cell Res. 2017 Mar 24;27(6):764–83. doi: 10.1038/cr.2017.41 (PMC5518870; doi:10.1038/cr.2017.41)
Supplement: Supplementary information, Table S3 — Mutations of target sequences in various CRISPR knockout MDA-MB-231 cancer cells [file cr201741x11.pdf]

Table S3. Mutations of target sequences in various CRISPR knockout MDA-MB-231 cancer cells

	**5`……3`**	Mutation
Casp3 KO	2528#: AAAGATCATACATGGAAGCGAAT-CAATGGACTCTGGAATAT<br>Casp3: AAAGATCATACATGGAAGCGAATTCAATGGACTCTGGAATAT	1bp deletion
Casp3/6 DKO (Based on Casp3 KO 2528#)	29118#: CACTGCC----AGGCGGGGCACCTGCGCAAATAGAGACACT<br>Casp6: CACTGCCAGAAAGGCGGGGCACCTGCGCAAATAGAGACACT	4bp deletion
Casp3/6/7 TKO (Based on Casp3/6 DKO 29118#)	TKO1#: GATGCTAAGCCACAC-GGTCCTCGTTTGTACCGTCCCTCTTCA<br>Casp7: GATGCTAAGCCAGACCGGTCCTCGTTTGTACCGTCCCTCTTCA	1bp deletion
EndoG KO	26013#: TGCCACCAACG-----TACCGCGGCAGTGGCTTCGACCGCG<br>EndoG: TGCCACCAACGCCGACTACCGCGGCAGTGGCTTCGACCGCG	5bp deletion
CAD KO	2945#: TGCGCAGCCCGAGGAAGTTCGG**G**CGTGGCTGGCCGGAGCTG<br>CAD: TGCGCAGCCCGAGGAAGTTCGG-CGTGGCTGGCCGGAGCTG	1bp insertion
EndoG/CAD DKO (Based on EndoG KO 26013#)	DKO12#: CCCT-TGCTGCCC**A**GAGGATGTTC----CCGCTGGCCGGAG<br>CAD: CCCTGCGCAGCCC-GAGGAAGTTCGGCGTGGCTGGCCGGAG	1bp insertion , 1+4 bp deletion
ATM KO	2561#: TCTAGATCGGCATTCAGATTCCAAA**A**CAAGGAAAATATTTGAA<br>ATM: TCTAGATCGGCATTCAGATTCCAAA-CAAGGAAAATATTTGAA	1bp insertion
ATR KO	2962#: AGCCAACCTCCGT**T**GATGTTGCTTGATTTCATCCAGCA<br>ATR: AGCCAACCTCCGT-GATGTTGCTTGATTTCATCCAGCA	1bp insertion
ATM/ATR DKO (Based on ATR KO 2962#)	DKO5#: GAAATTTAAGCGCCT-------GATCCTGAAACAATTAAACATCT<br>ATM : GAAATTTAAGCGCCTGATTCGAGATCCTGAAACAATTAAACATCT	7bp deletion

Note: Red: sgRNA sequence ; Yellow: PAM sequence; Bold: insertion sequence; -: deletion sequence. In all cases, knockout clones that showed both clear absence of target protein expression and gene mutations were chosen. In addition, in most cases, only those clones with homozygous mutations (where both copies of the gene showed the same mutation) were ease of identification. The only exception is in the case of endoG/CAD DKO, the mutations for endoG are heterozygous
