# Supplementary material for: Self-inflicted DNA double-strand breaks sustain tumorigenicity and stemness of cancer cells
Source: Cell Res. 2017 Mar 24;27(6):764–83. doi: 10.1038/cr.2017.41 (PMC5518870; doi:10.1038/cr.2017.41)
Supplement: Supplementary information, Table S4 — shRNA sequences of human JNK1 and JNK2 [file cr201741x12.pdf]

Table S4 . shRNA sequences of human JNK1 and JNK2

gene symbol	No.	clone ID*	sequence
JNK1	1	TRCN00 00010580	CCGGCCACAGAAATCCCTAGAAGAACTCGAGTTCTTCTAGGGATTTCTG TGGTTTTTG
	2	TRCN00 00010581	CCGGGACTCAGAACACAACAAACTTCTCGAGAAGTTTGTTGTGTTCTGA GTCTTTTTG
	3	TRCN00 00194860	CCGGCAGTAAGGACTTACGTTGAAACTCGAGTTTCAACGTAAGTCCTTA CTGTTTTTTG
JNK2	1	TRCN00 00000945	CCGGCTGTGAGGAATTATGTCGAAACTCGAGTTTCGACATAATTCCTCA CAGTTTTTG
	2	TRCN00 00010277	CCGGATCGTGAACTTGTCCTCTTAACTCGAGTTAAGAGGACAAGTTCAC GATTTTTTG
	3	TRCN00 00010280	CCGGGCGTCACCCATACATCACTGTCTCGAGACAGTGATGTATGGGTGA CGCTTTTTG
BAX	1	TRCN00 00033469	CCGGGATGTGGTCTATAATGCGTTTCTCGAGAAACGCATTATAGACCAC ATCTTTTTG
	2	TRCN00 00033470	CCGGGCCCACCAGCTCTGAGCAGATCTCGAGATCTGCTCAGAGCTGGT GGGCTTTTTG
BAK	1	TRCN00 00033464	CCGGCCTGTTTGAGAGTGGCATCAACTCGAGTTGATGCCACTCTCAAAC AGGTTTTTG
	2	TRCN00 00033465	CCGGCCGACGCTATGACTCAGAGTTCTCGAGAACTCTGAGTCATAGCGT CGGTTTTTG

*ID from Sigma-Aldrich catalog.
