# Supplementary material for: Self-inflicted DNA double-strand breaks sustain tumorigenicity and stemness of cancer cells
Source: Cell Res. 2017 Mar 24;27(6):764–83. doi: 10.1038/cr.2017.41 (PMC5518870; doi:10.1038/cr.2017.41)
Supplement: Supplementary information, Data S1 — Materials and Methods [file cr201741x13.pdf]

Supplementary information, Data S1 Materials and Methods

Source of cells

Breast cancer cell lines MDA-MB-231, MDA-MB-453, MDA-MB-468, MCF7, and colon cancer cell lines HT29, HCT116 were obtained from the Duke Cancer Institute Tissue Culture Core. They maintained in DMEM, supplemented with fetal bovine serum, and penicillin-streptomycin. MCF10A cells were also obtained the same place and maintained in DMEM/F12, supplemented with 5% horse serum, 20 ng/ml epidermal growth factor (EGF), 0.5 μg/ml hydrocortisone, 100 ng/ml cholera toxin, 10 μg/ml insulin, and penicillin-streptomycin. IMR90 cells were maintained in MEM, supplemented with 10% fetal bovine serum, and penicillin-streptomycin. The identities of the cells were verified by use of the STR (short tandem repeats) method by the Duke Cancer Institute Tissue Culture Core. None of the cell used are included in the database of commonly misidentified cell lines maintained by ICLAC.

Human patient-derived glioma cells T4121 and D456MG were kind gifts from Drs. Shideng Bao and Jeremy Rich of the Cleveland Clinic Learner Research Institute. They were isolated from patient-derived glioblastoma xenografts as previous described[1]. They were cultured in neural stem cell medium to keep their stem cell characteristics. The medium consists of neurobasal A medium (NBM, Invitrogen) with B27 supplement (Invitrogen), EGF (10 ng/ml, R&D), and fibroblast growth factor-basic (bFGF; 10 ng/ml, R&D). Cells were labeled with CD133/1(AC133) –phycoerythrin (PE) antibody kit (Miltenyi Biotec). CD133+ and CD133- cells were sorted or analyzed by flow cytometry.

Mycoplasma were tested in the cells by use of the Universal Mycoplasma Detection kit purchased from ATCC (Manassas, VA).

DNA damage assays

Synchronization protocol for DNA damage foci analysis: For analyzing γH2AX foci and 53BP1 foci, the cells were synchronized to G1 phase by low serum starvation. The main reason is to avoid the confounding observations of wide spread small γH2AX foci often present in S phase. In brief, the cells were plated at 25-30% confluency in a tissue culture dish, and changed to 0.1-0.5% FBS medium one day after plating. The cells were cultured with low serum for

another 3 days. EdU dye was used to identify S phase cells following the manufacture`s instruction of Click-iT EdU imaging kit (Invitrogen, Carlsbad, CA)

γH2AX Foci: γH2AX foci in cells were examined by use of an established protocol [2]. In brief, the cells were plated on glass-bottom 35-mm Petri dishes and treated with low serum to synchronize most of the cells into G1 phase. The cells were then were fixed, permeabilized, blocked, and then incubated with a primary γH2AX antibody overnight. After washing with cold PBS, cells were incubated with a secondary antibody conjugated with Alexa Fluor 488. Cells were then treated with mounting medium containing DAPI. Fluorescent images of γH2AX foci were acquired with a Leica SP5 confocal scanning microscope or Zeiss fluorescence microscope. The numbers of γH2AX foci were scored blinded from 5 randomly chosen fields (over 60 cells per field). Results were in most cases presented as the mean of three replicate experiments.

53BP1-mCherry reporter: To achieve live, non-invasive monitoring of DNA double strand breaks, we used a 53BP1-mCherry reporter (a kind gift from Dr. Titia de Lange of the Rockefeller University, New York, NY)[3]. The reporter was subsequently transferred into a lentiviral vector (pLex-53BP1-mCherry) in our laboratory. The 53BP1-mCherry reporter consists of a fusion between mCherry and a polypeptide domain representing amino acid 1220-1711 of 53BP1 protein. The fusion protein was shown to form foci in a similar manner as 53BP1. To observe 53BP1 foci, they were transduced with 53BP1-mCherry lentivector and selected with puromycin. The stably transduced cells were then sorted into different populations according to their mCherry fluorescence levels by use of FACS. Cells from different populations were then plated on glass-bottom 35 mm Petri dishes and synchronized into the G1 phase by low serum starvation. Fluorescent images of 53BP1-mCherry were acquired with a Leica SP5 confocal scanning microscope or Zeiss fluorescence microscope. 53BP1-mCherry foci were then scored in a blinded manner.

Neutral comet assay: The comet assay (single cell gel electrophoresis) was used to detect cells with DNA double strand breaks[4]. It was performed by use of a commercially purchased kit from Trevigen (Gaithersburg, MD). Manufacturer`s procedures were followed. Cells were harvested and mixed into liquid phase low melting agarose and layered onto the slides and lysed following

manufacture's protocol. For electrophoresis of the DNA, an electric current of 21 Volts was applied for 40 min neutral electrophoresis buffer. After electrophoresis, the slides were stained with SYBR-Gold and imaged by use of a Zeiss fluorescence microscope. The percentage of DNA in comet tail, which reflects DNA damages of host cells was analyzed by use of Image J software (NIH). Results were presented as the mean of three independence experiments.

Cytochrome c leakage from the mitochondria

Cytochrome c is localized in the mitochondrial intermembrane space under normal physiological conditions. The release of cytochrome c from mitochondria to cytosol often happens during MOMP (mitotochondria outer membrane permeabilization). Cytochrome c leakage was detected by use of two methods. In the first method, we analyzed the cytosolic cytochrome c in various cell lines by western blot. To obtain the cytosolic fraction a kit from Abcam (Cambridge, MA) was used. Cells were lysed and centrifuged following manufacturer`s procedures. The supernatant (cytosol) and pellets (mitochondria) were stored at –70 °C for western blot analysis. In the second method, we detected the distribution of cytochrome c in different cells by immunofluorescence staining.

CRISPR/Cas9-mediated gene knockout

We make various tumor cells deficient in various genes by use of the CRISPR/Cas9 technology. Single guided RNA (sgRNA) sequences targeting the genes were generated with the use of a free online CRISPR design tool (crispr.mit.edu). The sgRNA sequences used were listed in **Supplementary Table S2**. Annealed double stranded sgRNA oligos were ligated into the lentiCRISPR vector [5] (deposited by Dr. Feng Zhang to Addgene, Cambridge, MA) at BsmBl site, which co-express cas9 and sgRNA in the same vector. The constructed CRISPR lentivirus vectors were then packaged according to a standard protocol. To produce lentiviral vectors, lentiviral plasmids with the target genes were transduced into 293T cells together with second generation packaging plasmids (psPAX2, pMD2.G) following previously published procedures: http://tronolab.epfl.ch/lentivectors.

Subsequently, tumor cells were infected with CRISPR lentivirus vector and selected in purimycin for 14 days. The infected cells were then plated to 96-well plates at 1 cell per well.

When the clonal cell populations became visible, they were transferred and expanded for western blot detection. Those clones that showed no target protein expression were then subjected to PCR (around sgRNA target sequence) and Sanger sequencing verification of gene disruption. To generate double knockout cells (with knockout of a second gene), those with the first gene knockout were infected a second sgRNA lentivirus vector (lentiCRISPR-sgRNA-BFP) and cultured for 14 days. Clonal cell populations were screened for target gene deficiency following the same procedure. For knockout of the ATM gene in glioma cells, we used a multiplex sgRNA vector obtained from Dr. Charles Gersbach [6].

ShRNA mediated gene knockdown

For knockdown of JNK1, and JNK2 gene expression in MDA-MB-231 cells, we obtained pLKO.1 lentiviral vector from Sigma-Aldrich (St. Louis ,MO) encoding shRNA minigene against JNK1 or JNK2 gene. The shRNA sequences used were listed in Supplementary Table S4. Knockdown efficiency was determined by use of Western blot analysis.

Western blot analysis

Cells were lysed in RIPA lysis buffer (SIGMA, St Louis, MO) with protease inhibitor (Millipore, Billerica, MA). Samples were separated by SDS-PAGE and transferred to PVDF membranes (Bio-Rad, Hercules, CA). The membranes were then blotted with primary and secondary antibodies. Detailed information for all antibodies is listed in **Supplementary Table S1**. Signal was detected using enhanced chemiluminescence reagent (Thermo Scientific, Rockford, IL).

Soft agar assay

Tumor cells were plated into 6-well plates with 1.5 ml 0.3%(w/v) low melting agar (BD-Sparks, MD), which was overlaid onto 1.5 ml 0.6% (w/v) bottom agar layer. Soft-agar cultures were maintained at 37°C for 3 weeks. The colonies were then stained with 0.005% crystal violet. The colonies were counted by use of Image J software (NIH). Results were presented as the means of three independent experiments.

ELISA

To measure interleukin 6 (IL-6), and interleukin 8 (IL-8) present in the supernatant of vector-transduced and knockout MDA-MB-231 cells, 100,000 cells were plated in each well of 6-well plates with 2 ml medium. The supernatant was collected at 48h. The IL-6 and IL-8 were then measured by use of the Quantikine ELISA Kit (R&D, Minneapolis, MN) according to manufacturer's instructions

Artificial induction of DNA double strand breaks and tumor formation

Nucleus-targeted EndoG system: Endonuclease G (EndoG) is a nuclease that can generate double strand breaks during apoptosis. EndoG naturally resides in the mitochondria due to a presence of a mitochondrion targeting signal (1-48 aa). To re-target EndoG into the nuclear so it can generate DSBs in target cells, a nuclear localization signal (NLS, 5'GGCCCAAAGAAGAAGAGAAAGGTT3' or GPKKKRKV in amino acid sequence) from the SV40 large T antigen was fused to the N-terminal end of a truncated EndoG domain representing amino acid 49-297 (thus missing its native mitochondria targeting signal). The NLS-EndoG cassette was then inserted into a lentivirus vector.

Immunofluorescence analysis

Cells were cultured on glass-bottom 35mm petri dishes. Cells were fixed with 4% paraformaldehyde (PFA) in PBS for 15min, permeabilized and blocked with PBS containing 5% donkey serum, 0.1% Triton X-100 and 1% BSA for 45 min. Fixed cells were incubated with primary antibodies in 1% BSA overnight at 4°C, followed by incubated with appropriate Alexa Fluor 488,or 555-conjugated secondary antibodies (Invitrogen, Carlsbad, CA) for 1 h and mounted with mounting medium (Vector Laboratories, CA) containing DAPI. See **Supplementary Table S1** for information on the antibodies. Fluorescent images were acquired using Lecia SP5 confocal scanning microscope.

Limited dilution analysis and tumor sphere formation

Glioma cells were serially diluted with neural stem cell medium and plated into 96-well plates at a final cell number per well of 1, 2, 5, 10, 20, 50, 100, or 200. Tumor sphere formation was

evaluated 21 days after plating and wells were scored positive for the presence of at least one tumor sphere. The analysis was conducted according to previously published methods [7, 8].

Tumor xenografts

All animal experiments conducted in this study were approved by the Duke University Institutional Animal Care and Use Committee. Six weeks old female athymic Balb/C nude mice were obtained from Jackson Laboratories (Bar Harbor, Maine). Vector-transduced MDA-MB-231 cells or CRISPR/CAS9 knockout cells (2×10^5 cells in 50 µl of sterile PBS) were injected subcutaneously into the flanks of nude mice (n=6/group). MCF-7 and MCF-7-CASP3 tumor cells ($3x10^6$ in 50µl of sterile PBS) were injected subcutaneously with matrigel by use of female nude mice (n=10) implanted with estrogen pellets (1.7 mg/pellet, 60 day release formula, Innovative Research of American). T4121 and D456MG glioma cells($1x10^6$ in 50µl of sterile PBS) were injected subcutaneously with matrigel by use of female severe combined immunodeficiency (SCID) mice (n=6) . After inoculation, the growth of tumors was evaluated every 3 days by use of a caliper.

The animal numbers in each group were decided based on our prior experience with the tumor model based on the criteria that we want to achieve a power of 0.80 with significance level of $p<0.05$. Animals were only excluded from analysis if they died during experiments. Animals were randomized according to their body weights. The technician conducting tumor cell injection and measurements were blind to the cell identities.

Analysis of patient-derived tumor specimens

Pre-treatment samples of breast and colon cancer were obtained from patients when they undergo treatment at Cancer Center, Shanghai General Hospital, Shanghai Jiaotong University School of Medicine. The clinical pathological characteristics of breast cancer and colon cancer patients were summarized in Supplementary Tables S5 and S6, respectively. All studies were carried out with the approval of the Medical Ethics Committee of Shanghai General Hospital. Informed consent was obtained for all patients treated since 2010.

For immunohistochemistry staining, the tissue slides were deparaffinized first. Antigen retrieval was then carried out and the slides were then incubated with primary antibodies overnight at 4 °C, followed by use of secondary antibody(Gene Tech, Shanghai, China) for 30 min at room temperature. DAB (3,3-diaminobenzidine) was used to visualize positive immune reaction. Nuclei were counterstained with hematoxylin. The primary antibodies included rabbit anti-pATM (S1981), and rabbit anti-cleaved caspase3 (CC3). pATM was scored and sorted into 3 categories based on staining extent (negative for no staining or uncertain staining, ≤25 % positive staining for low expression, >25% positive staining for high expression). CC3 was scored and put into 3 categories based on staining extent (negative for no staining or uncertain staining, ≤10 % positive staining for low expression, >10 % positive staining for high expression).

For immunofluorescence co-staining, breast cancer tissue slides with high expression of CC3 were co-staining with primary antibodies overnight at 4 °C, followed by incubated with appropriate Alexa Fluor 488, or 555-conjugated secondary antibodies (Invitrogen, Carlsbad, CA) for 1 h at room temperature and mounted with mounting medium (Vector Laboratories, CA) containing DAPI. The pairs of primary antibodies included rabbit anti-pATM /mouse anti-CC3, rabbit anti-pNEMO /mouse anti-CC3, rabbit anti-pSTAT3 Y705 /mouse anti-CC3, rabbit anti-pATM /mouse anti-EndoG. Fluorescent images were acquired by use of a Lecia SP5 confocal scanning microscope.

Statistical analysis

Sample sizes in most experimental groups were decided based on the criteria to obtain a power of 0.80 to achieve *p* values of 0.05 or smaller. Statistical analyses were performed by use of the SPSS software. For most of *in vitro* and animal experiments, two-sided unpaired Student's t test was used to calculate the p-values. Values of $p < 0.05$ were considered statistically significant. Survival curves were plotted using Kaplan-Meier method, and analyzed using the log-rank test. Actual statistical tests are described either in the figure legends or relevant places in the text. Estimate of variance were done for all data points involving multiple replicates.

References

1 Bao S, Wu Q, McLendon RE *et al.* Glioma stem cells promote radioresistance by preferential activation of the DNA damage response. *Nature* 2006; **444**:756-760.

2 Rogakou EP, Pilch DR, Orr AH, Ivanova VS, Bonner WM. DNA double-stranded breaks induce histone H2AX phosphorylation on serine 139. *J Biol Chem* 1998; **273**:5858-5868.

3 Dimitrova N, Chen YC, Spector DL, de Lange T. 53BP1 promotes non-homologous end joining of telomeres by increasing chromatin mobility. *Nature* 2008; **456**:524-528.

4 Olive PL, Banath JP, Durand RE. Detection of etoposide resistance by measuring DNA damage in individual Chinese hamster cells. *J Natl Cancer Inst* 1990; **82**:779-783.

5 Cong L, Ran FA, Cox D *et al.* Multiplex genome engineering using CRISPR/Cas systems. *Science* 2013; **339**:819-823.

6 Kabadi AM, Ousterout DG, Hilton IB, Gersbach CA. Multiplex CRISPR/Cas9-based genome engineering from a single lentiviral vector. *Nucleic Acids Res* 2014; **42**:e147.

7 Bellows CG, Aubin JE. Determination of numbers of osteoprogenitors present in isolated fetal rat calvaria cells in vitro. *Dev Biol* 1989; **133**:8-13.

8 Tropepe V, Sibilia M, Ciruna BG, Rossant J, Wagner EF, van der Kooy D. Distinct neural stem cells proliferate in response to EGF and FGF in the developing mouse telencephalon. *Dev Biol* 1999; **208**:166-188.
